# The novel tumour suppressor Madm regulates stem cell competition in the *Drosophila* testis

**DOI:** 10.1038/ncomms10473

**Published:** 2016-01-21

**Authors:** Shree Ram Singh, Ying Liu, Jiangsha Zhao, Xiankun Zeng, Steven X. Hou

**Affiliations:** 1 The Basic Research Laboratory, National Cancer Institute, National Institutes of Health, Frederick, Maryland 21702, USA

## Abstract

Stem cell competition has emerged as a mechanism for selecting fit stem cells/progenitors and controlling tumourigenesis. However, little is known about the underlying molecular mechanism. Here we identify Mlf1-adaptor molecule (Madm), a novel tumour suppressor that regulates the competition between germline stem cells (GSCs) and somatic cyst stem cells (CySCs) for niche occupancy. *Madm* knockdown results in overexpression of the EGF receptor ligand *vein* (*vn*), which further activates EGF receptor signalling and integrin expression non-cell autonomously in CySCs to promote their overproliferation and ability to outcompete GSCs for niche occupancy. Conversely, expressing a constitutively activated form of the *Drosophila* JAK kinase (*hop^Tum−l^*) promotes Madm nuclear translocation, and suppresses *vn* and integrin expression in CySCs that allows GSCs to outcompete CySCs for niche occupancy and promotes GSC tumour formation. Tumour suppressor-mediated stem cell competition presented here could be a mechanism of tumour initiation in mammals.

## Abstract (web summary)

Stem cell competition mediates the balance between tissue homeostasis and tumour formation, but how this occurs is unclear. Here, Singh *et al.* show that the tumour suppressor Mlfl-adaptor molecule regulates the balance between germline stem cell and somatic cyst stem cell growth in the Drosophila testis niche.

## Graphical abstract

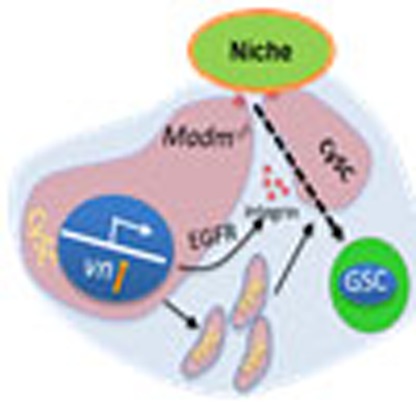

Animal tissues and organs are generated and maintained by stem cells, which generate most of the cell types that form an organ during development. These stem cells maintain tissue homoeostasis by supplying new cells to replace dying or damaged ones in adult animals. Recent studies suggest that Mother Nature selects the fittest stem cells for tissue development and homoeostasis through stem cell competition^1^,^2^,^3^,^4^,^5^,^6^,^7^. Stem cell competition has also been shown to be a mechanism for both tumour suppression^4^ and tumour initiation^1^,^8^. However, little is known about the underlying molecular mechanism.

*Drosophila* testis germline stem cells provide one of the best genetic systems for studying stem cell niche interactions at the cellular and molecular levels^2^,^9^,^10^,^11^,^12^,^13^,^14^,^15^,^16^,^17^,^18^,^19^,^20^,^21^,^22^,^23^,^24^,^25^,^26^,^27^,^28^,^29^,^30^,^31^,^32^,^33^. At the tip of the *Drosophila* testis (apex) is a germinal proliferation centre, which contains the germline stem cells (GSCs) and somatic stem cells (CySCs) that maintain spermatogenesis (Fig. 1a)^34^,^35^,^36^. Each GSC is encysted by two CySCs. Both the GSCs and CySCs anchor to a group of 12 non-dividing somatic cells, called the ‘hub'^37^, through cell-adhesion molecules^31^,^38^. The hub defines the stem cell niche by expressing the growth factor Unpaired (Upd), which is the ligand that activates the JAK-STAT pathway in adjacent GSCs and CySCs to regulate their self-renewal. The ratio of GSCs to CySCs in a wild-type testis niche is 1:2 and is precisely coordinated by differentially regulating JAK-STAT signalling levels in the two different stem cell types^2^,^25^. In addition, several other signalling pathways regulate the behaviour of these two stem cells at the testis niche^20^,^21^,^39^,^40^,^41^,^42^,^43^.

In a genetic screen for mutations that regulate male GSC fates, we identified Mlf1-adaptor molecule (Madm)^44^,^45^. Myeloid leukaemia factor 1 (Mlf1) is an oncogene and is originally described by a translocation between *Mlf1* on chromosome 3 and nucleophosmin (*NPM*) on chromosome 5, which yields the oncogene *NPM-MLF1* (ref. 46). Madm and 14-3-3ζ were identified as binding partners of Mlf1 in a yeast two-hybrid screen^45^. The expression of exogenous Mlf1 potentiated M1 maturation, while ectopic expression of *Madm* in M1 myeloid cells suppressed cytokine-induced differentiation. The opposing effects of Madm and Mlf1 on M1 cell differentiation suggest that Madm may function as a tumour suppressor. In humans, Madm was also named a nuclear receptor binding protein 1 (NRBP1) (ref. 47). Just as *H37N21.1* (*NRBP1*) knockdown cooperates with gain-of-function mutations in *let-60* (*Ras*) to regulate vulval formation in *Caenorhabditis elegans*, *NRBP1* knockdown cooperates with a constitutively activated form of *Ras* (*Ras*^*V12*^) in the transformation of mammalian cells in culture^48^. Somatic loss of *Nrbp1* in mice results in tumourigenesis, with haematological and intestinal tumours predominating. The NRBP1 protein is downregulated in a wide variety of human tumours, and survival data analysis indicates that low expression correlates with poor prognosis. These data together suggest that *NRBP1* is a conserved regulator of cell fate and plays an important role in tumour suppression^48^.

In this study, we show that *Madm* is specifically expressed in CySCs and coordinates with the JAK-STAT and EGFR signal transduction pathways to regulate stem cell competition in the *Drosophila* testis.

## Results

### Madm functions in CySCs to guide GSCs-CySCs competition

To identify new stem cell regulators in the *Drosophila* testis, we carried out a screen in which a collection of transgenic RNAi lines^49^,^50^,^51^ were crossed with *act-Gal4; tub-Gal80*^*ts*^ flies (referred to as *Act*^*ts*^). *act-*Gal4 is ubiquitously expressed in the *Drosophila* testis (Supplementary Fig. 1a–c). The adult flies were shifted to the restrictive temperature (29 °C) from 18 °C and cultured for different times. The flies were then dissected, stained and examined for GSCs under confocal microscopy. One of the first few genes identified in this screen was *Madm*. *Madm* knockdown by transgenic RNAi (*Madm*^RNAi^, BL31644) resulted in a dramatic decrease of GSCs in the testes compared with that of wild-type flies (Supplementary Fig. 1d–g). Knockdown of genes by dsRNAs often produce false-positive phenotypes because of off-target effects^52^. We ruled out the possibility of false-positive effects and confirmed the *Madm* phenotype with three other transgenic RNAi lines (v27346, BL41599 and BL42529) generated from independent sequences^49^,^51^ (Supplementary Fig. 1h,i).

To further understand the function of Madm in the germline or in the soma, we knocked down *Madm* by cell-type-specific Gal4s and *UAS-Madm*^RNAi^ transgenic fly lines. We found that depleting *Madm* in germ cell lineage using *nanos* (*nos*)*-Gal4* (*Nos*>*Madm*^RNAi^; Supplementary Fig. 1j), and in the hub cells using *unpaired* (*upd*)*-Gal4* (*Upd*>*Madm*^RNAi^; Supplementary Fig. 1k) resulted in no obvious phenotype. We also generated negatively marked GSC clones of wild-type or *Madm*^44^ flies using the FLP/FRT mosaic analysis technique^53^ and found that *Madm* is not required in GSCs for their maintenance (Supplementary Fig. 2a–c). These data together suggest that *Madm* is not required in the germ cell lineage and hub cells.

**Figure 1:**
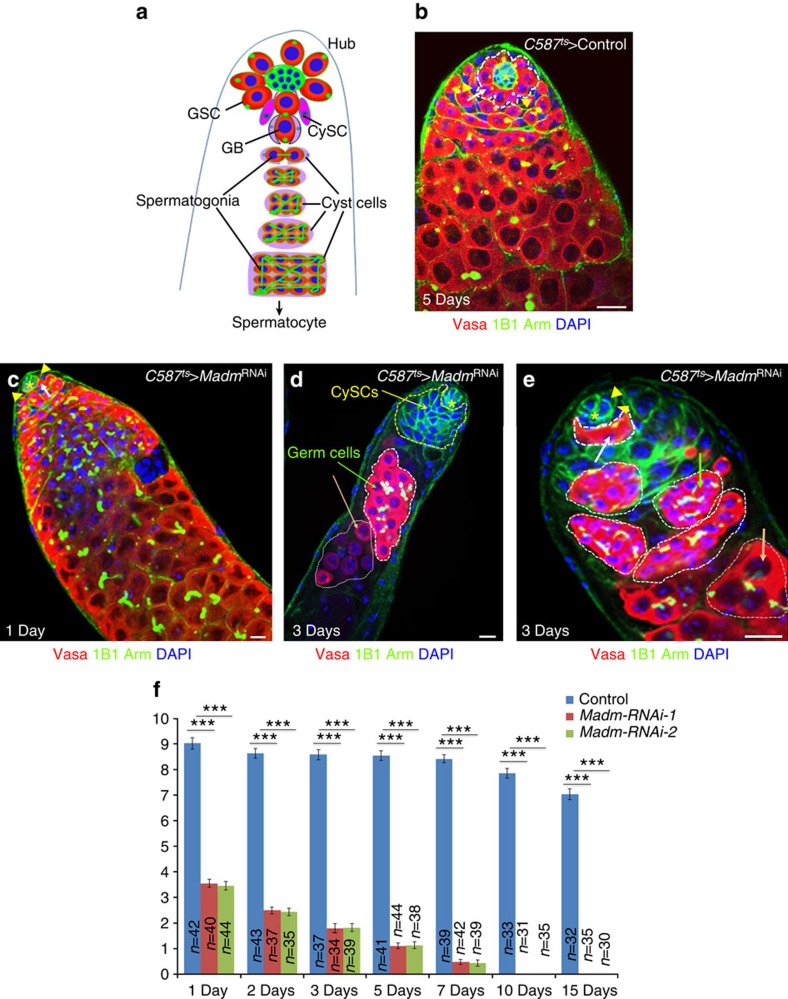
Madm functions in CySC to regulate GSC maintenance. (**a**) A schematic diagram of *Drosophila* adult testis. GSCs and GBs (gonialblasts) contain the spherical spectrosomes (green dots), while spermatogonia and spermatocytes contain branched fusomes (green lines). GSCs are encysted by two CySCs (magenta). GBs and other germ cells (red) are encapsulated by cyst cells (lavender). (**b**–**e**) The confocal section of the testis apex from *C587*^ts^*>*control (**b**), and *C587*^ts^*>Madm*^RNAi−1^ (BL31644; **c**–**e**) males after shifting the temperature from 18° to 29 °C for 1 to 15 days as adult flies. In control testis (**b**, 5 days at 29 °C, *n*=41) contain a rosette of GSCs around the hub (white dotted lines with one GSC is highlighted by an arrow), and CySCs are located away from the hub (yellow arrowhead). In *C587*^ts^*>Madm*^RNAi^ testes (**c**, 1 day, *n*=40; (**d**,**e**), 3 days, *n*=39), the number of GSCs are gradually lost from the niche and differentiated (highlighted by white dotted lines in **d**,**e**), and CySCs moved into the niche (highlighted by yellow dotted lines in **d**). The testes were stained with antibodies against Vasa (red, marks all germ cells including GSCs), 1B1 (green, marks spherical spectrosomes and branched fusomes), Arm (green, hub cells at the tip). DAPI (blue) stains nuclei. Asterisks indicate hub cells. White arrows near hub indicate GSC. Green arrows (in **d**,**e**) and orange arrow (**d**,**e**) away from hub with white dotted lines indicate spermatogonial cells and spermatocytes, respectively. (**f**) A bar graph showing the number of GSCs per testis in *C587*^ts^>control, *C587*^ts^*>Madm*^RNAi−1^ (BL31644) and *C587*^ts^*>Madm*^RNAi−2^ (v27346) flies. Flies were cultured from 1 to 15 days at 29 °C before staining with antibodies against Vasa, 1B1 and Arm. Statistical significance determined by Student's *t*-test, ***P<0.0001. All values are mean±s.e.m. Scale bar, 10 μm (**b**–**e**).

We further knocked down *Madm* in the CySC lineage using *C587-Gal4* (refs 14, 15, 30; *C587*^ts^>*Madm*^RNAi^; Fig. 1b–e) and found that the GSCs, then spermatogonia and finally spermatocytes were lost over time at 29 °C (Fig. 1c–f and Supplementary Fig. 3a–h) similar to the phenotypes of *Act*^ts^>*Madm*^RNAi^ flies (Supplementary Fig. 1d–g,i), suggesting that *Madm* functions in the CySC lineage to regulate GSC maintenance. Further, dying GSCs were not detected in wild-type and *Madm*^RNAi^ testes, and similar numbers of dying spermatogonial cysts were detected in both control and *Madm*^RNAi^ testes (Supplementary Fig. 4a–c).

**Figure 2:**
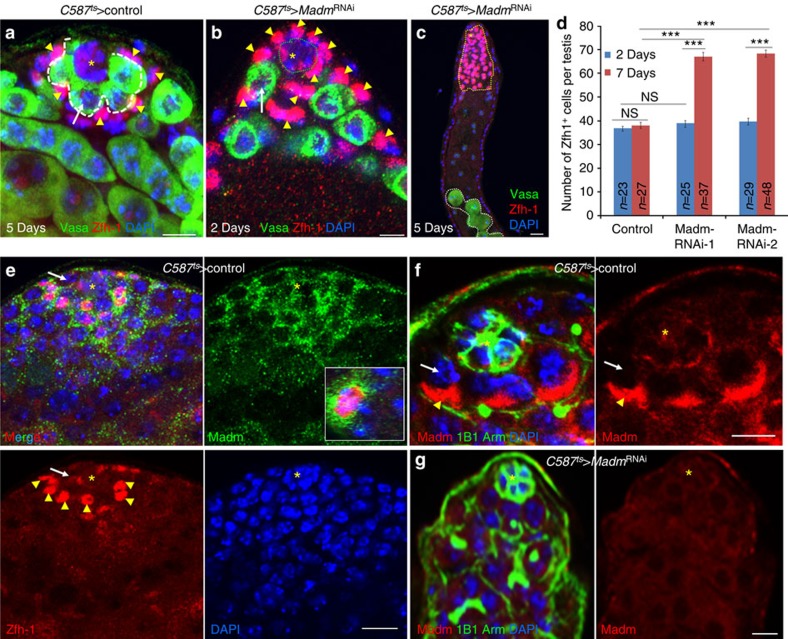
Madm functions in CySCs to prevent CySCs from outcompeting GSCs for niche occupancy. (**a**–**c**) A confocal section of the testis apex containing *C587*^ts^>control (**a**) and *C587*^ts^*>Madm*^RNAi^ (BL31644) (**b**, 2 days, *n*=25; **c**, 5 days, *n*=30) males after shifting the temperature from 18^o^ to 29 °C for 2, 5 and 7 days as adult flies. The testes were stained with antibodies against Vasa (green, marks all germ cells including GSCs), Zfh-1 (red, marks CySCs and their immediate cyst cell daughters). DAPI (blue) stains nuclei. (**d**) A bar graph showing the number of Zfh-1+ cells per testis in *C587*^ts^*>*control, and *C587*^ts^*>Madm*^RNAi−1^ (BL31644) and *C587*^ts^*>Madm*^RNAi−2^ (v27346) flies at 2 and 7 days at 29 °C. Statistical significance determined by Student's *t*-test, ***P<0.0001; NS indicates not significant (*P*>0.05). All values are mean±s.e.m. (**e**–**g**) The testes of *C587*^ts^*>*control (**e**) were stained with Zfh-1 (red) and Madm (green), highlighted in inset a co-stained of Zfh-1 and Madm. The testes of *C587*^ts^*>*control (**f**) and *C587*^ts^*>Madm*^RNAi−1^ (**g**) were stained with Madm (red), 1B1 and Arm (green), DAPI (blue). *indicate hub cells. GSCs are highlighted by white arrows and white dotted lines near hub cells in **a** and **b**, and by white arrows in **e** and **f**. Differentiated germ cells (away from the hub) in **c** are highlighted by white dotted line (green arrow). CySCs are highlighted by arrowhead (yellow) in **a**,**b**,**e**,**f**. Yellow dotted line in **c** highlighted Zfh-1-positive cells. Scale bar, 10 μm (**a**–**c**, **e**–**g**).

In the *Drosophila* testis, GSCs and CySCs share the same niches. We further investigated CySCs in *Madm* knockdown testes by examining the expression of the zinc-finger homeodomain-1 (Zfh-1) protein, which marks CySCs and their immediate cyst cell daughters^19^ (Fig. 2a–c). In the wild-type testes, the nuclei of Zfh-1-positive CySCs are farther from the hub than the nuclei of GSCs (Fig. 2a, arrowhead). In the *C587*^ts^>*Madm*^RNAi^ testes, after shifting to the restrictive temperature, the Zfh-1-positive CySCs gradually moved into the niches and pushed the GSCs out (Fig. 2b,c, arrowhead) and the number of Zfh-1-positive cells were significantly (Student's *t*-test, *P*<0.0001) increased over time (Fig. 2d). Therefore, the normal function of Madm is to prevent CySCs from outcompeting GSCs for niche occupancy.

Using antibodies to Madm^44^, we detected Madm expression in the somatic cells of the wild-type testes, particularly in CySC cytoplasm (Fig. 2e,f); while the expression of Madm was markedly reduced in the *C587*^ts^>*Madm*^RNAi^ testes (Fig. 2g), suggesting that Madm protein expression was effectively knocked down in the *C587*^ts^>*Madm*^RNAi^ testes. These data together suggest that Madm functions in CySCs to regulate CySCs and GSCs competition for niche occupancy.

### The outcompeted GSCs became differentiated rather than died

We further investigated the fate of the outcompeted (loser) GSCs. First, we found that flies that had been kept in 29 °C for 3 days lacked GSCs but contained spermatogonia (Supplementary Fig. 3c,d,h), indicating that the outcompeted GSCs possibly became differentiated. Second, we examined cell death with TUNEL labelling of the *C587*^ts^>*Madm*^RNAi^ flies after culturing at 29 °C for 3 or 6 days and did not find any significant (Student's *t*-test, *P*>0.05) increase of GSC death in comparison with that of the *C587*^ts^>control flies (Supplementary Fig. 4a–c). Third, we wondered whether testes from *Madm*^RNAi^ flies lost the GSCs at 29 °C (Fig. 1c–f and Supplementary Fig. 3a–h) could restore them after recovery at 18 °C. It has been demonstrated that differentiating spermatogonial cysts revert to stem cell identity when signalling is restored^10^. We fed bromodeoxyuridine to flies that had been kept in 29 °C for 3 days. These flies lacked GSCs, but contained spermatogonia (Supplementary Fig. 3h). Labelled CySCs were detected near the hub (arrowhead), while spermatogonial cysts (4, 8 and 16 cells) were detected away from the hub before recovery (Supplementary Fig. 5a,b). However, after 2 days of recovery at 18 °C, breakdown of spermatogonial cysts were detected and 14.15% of testes regained labelled GSCs (Supplementary Fig. 5c,h). After 4 days of recovery, 47.3% of testes regained labelled GSCs (Supplementary Fig. 5d,e,h). We compared the percentage of testes containing any GSCs before and after recovery, and found that after 2 days of recovery only 14.15% of testes contained any GSCs; however, 84.2% of testes contained GSCs after 6 days of recovery at 18 °C (Supplementary Fig. 5f–h). These data together suggest that the outcompeted GSCs became differentiated and can revert back to GSCs when the Madm activity is restored.

### Madm non-cell autonomously directs CySC-GSC competition

**Figure 3:**
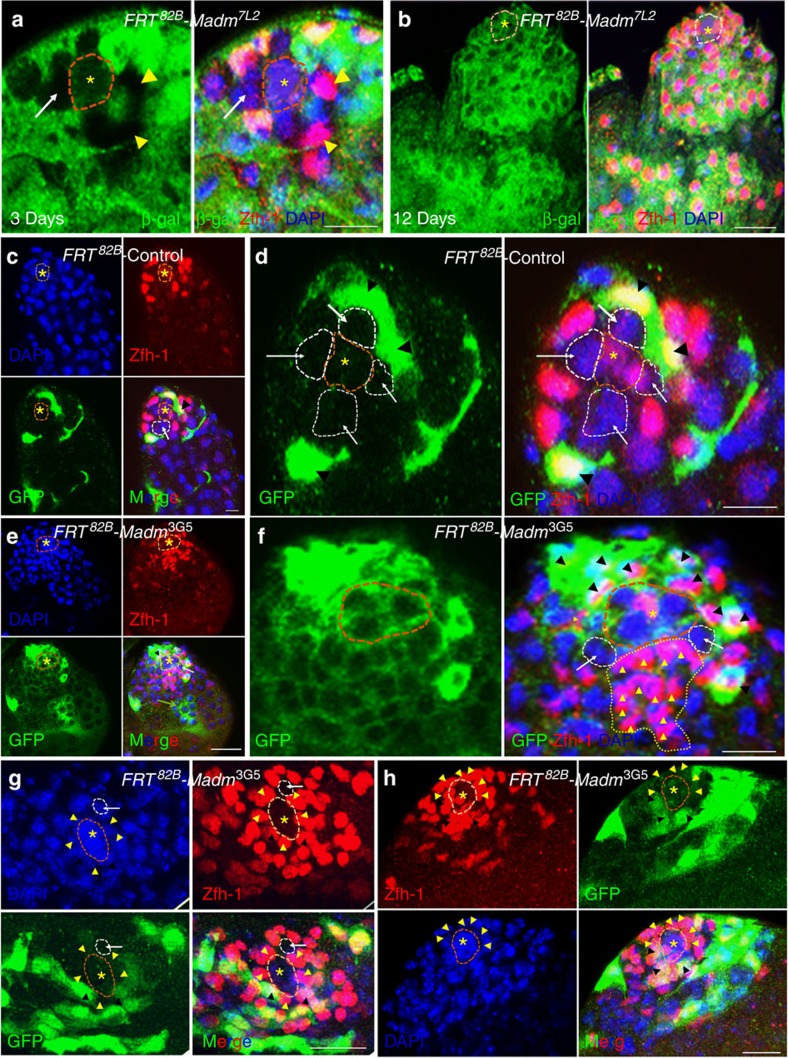
Madm functions in CySC non-cell autonomously and regulates the competition between GSCs and CySCs. (**a**,**b**) Confocal sections through the apex of the testes containing *FRT*^*82B*^-*Madm*^7L2^ clones at 3 days ACI (a, *n*=45) and 12 days ACI (b, *n*=52). The testes were stained with the Zfh-1 (red), β-galactosidase (green), and DAPI (blue). The *FRT*^*82B*^*-Madm*^7L2^ CySC clones are β-galactosidase (green) negative and Zfh-1 (red) positive. β-galactosidase negative CySCs clones are highlighted by yellow arrowhead (**a**,**b**). (**c**–**h**) GFP^+ve^ clones were generated in the testes of wild-type control (*FRT*^82B^*-PiM*; **c**,**d**, *n*=56) or *FRT*^82B^*-Madm*^3G5^ (**e**–**h**, *n*=62) flies using the MARCM technique, and were stained at 7 days ACI with GFP (green), Zfh-1 (red) and DAPI (blue). GFP-positive CySCs clones are highlighted by black arrowheads (**c**–**h**). GFP-negative CySCs clones are highlighted by yellow arrowheads (**e**–**h**). Green arrow in **e** indicates differentiated germ cells. Asterisks with orange dotted lines indicate hub cells. GSCs are highlighted by white arrows and white dotted lines near hub cells. Scale bar, 10 μm (**a**–**h**).

To further examine the function of Madm in CySCs, we generated LacZ (*arm-lacZ*)-negative CySC clones of wild-type or *Madm*^44^ flies using the FLP/FRT mosaic analysis technique^53^ and analyzed the phenotypes 3 and 12 days after clone induction (ACI) (Fig. 3a,b and Supplementary Fig. 6a). We uncovered a unique role of *Madm* in CySCs. Both wild-type and *Madm* mutant testes initially contain only a few LacZ-negative and Zfh-1-positive CySCs (Fig. 3a, and Supplementary Fig. 6a) at 3 days ACI. However, LacZ-positive and Zfh-1-positive CySCs were significantly increased and occupied the niche in the *Madm* mutant testes (Fig. 3b and Supplementary Fig. 6a), while both LacZ-negative and LacZ-positive CySCs in wild-type testes remained relatively constant at 12 days ACI^2^ (Supplementary Fig. 6a). We also generated GFP positively marked CySC clones of wild-type or *Madm* flies using the mosaic analysis with a repressible cell marker (MARCM) technique^54^ and analyzed CySC lineages 7 days ACI (Fig. 3c–d). Consistent with our previous mosaic analysis results, we found that the GFP-negative and Zfh-1-positive CySCs were significantly (Student's *t*-test, *P*<0.0001) increased and occupied the niche in the *Madm* mutant testes (Fig. 3e–h and Supplementary Fig. 6b,c) compared with those in the wild-type testes (Fig. 3c,d and Supplementary Fig. 6c). Further, by staining testes with either wild-type or *Madm* mutant MARCM clones with GFP and Vasa, we found two unique phenotypes: (i) GFP-positive CySCs directly attaching to hub cells in *Madm* mutant testes while such cells locating outside GSCs in wild-type testes and (ii) only one Vasa-positive GSC left at the niche in the *Madm* mutant testes while normal number (5–9) of GSCs at the niche in the wild-type testes (compare Supplementary Fig. 6e–g with Supplementary Fig. 6d). These analyses together suggest that CySCs have pushed GSCs away from the niche. We further examined the mitotic index in wild-type or *C587*^ts^>*Madm*^RNAi^ testes (Supplementary Fig. 6h–j) by staining with anti-phospho-Histone H3 (pH3) and found that the pH3-positive somatic cells were significantly (Student's *t*-test, *P*<0.0001) increased in the *C587*^ts^>*Madm*^RNAi^ testes (Supplementary Fig. 6i,j) compared with those in the wild-type testes (Supplementary Fig. 6h,j). These data together suggest that *Madm* non-cell autonomously regulates CySC proliferation and the ability of CySCs to outcompete GSC for niche occupancy.

### Madm non-cell autonomously regulates integrin and p-dERK

It was reported that SOCS36E suppresses JAK-STAT signalling specifically in the CySCs, preventing them from displacing neighbouring GSCs, by upregulating the adhesion protein integrin^2^,^25^. The function of *Madm* is somehow similar to that of SOCS36E. The expression of Stat92E is regulated by JAK-STAT signalling and the level of Stat92E protein reflects JAK-STAT signalling activity^55^. To investigate the function of *Madm* on JAK-STAT signalling, we examined Stat92E protein expression in wild-type and *Madm* knockdown testes (*C587*^ts^>*Madm*^RNAi^) (Supplementary Fig. 7a–d). In wild-type testes, Stat92E is specifically enriched in GSCs, while CySCs, hub cells and all differentiated cells express little Stat92E, indicating that GSCs normally have higher JAK-STAT signalling than that of CySCs (Supplementary Fig. 7a,e). In *Madm* knockdown testes (*C587*^ts^>*Madm*^RNAi^; Supplementary Fig. 7b–d), CySCs and hub cells have elevated levels of Stat92E (Supplementary Fig. 7e). This result suggests that *Madm*, functioning similarly to SOCS36E, normally attenuates JAK-STAT signalling in CySCs and hub cells to maintain the appropriate balance of the two stem cell lineages in the niche.

We further examined the expression of the common β-subunit of position-specific integrins (βPS-integrin) in wild-type and *Madm* knockdown testes (*C587*^ts^>*Madm*^RNAi^; Supplementary Fig. 8a–f). The βPS-integrin level is significantly (Student's *t*-test, *P*<0.0001) higher in *Madm* knockdown testes (Supplementary Figs 8c,d,g) than in wild-type testes (Supplementary Fig. 8a,b,g), particularly at the CySC–hub interface (Supplementary Fig. 8c,f). We also examined the expression of βPS-integrin together with DE-cadherin (mark hub cells) and found that integrin is not incorporated in hub cells, but rather highly expressed at the hub-CySC interface in *Madm* knockdown testes (Supplementary Fig. 8f). The number and appearance of hub cells were unaffected in *Madm* knockdown testes as compared with wild-type testes (Supplementary Fig. 8e,f; Control: 22.83±0.41, *n*=12, versus *Madm*^RNAi^: 24.14±0.55, *n*=14, hub cells per testis, *P*>0.05). This result indicates that *Madm* may regulate stem cell competition by controlling integrin expression. To further test this hypothesis, we examined the genetic interaction between *Madm* and *rhea*, an integrin-binding cytoskeletal linker that is essential for integrin-mediated adhesion^56^. Removing one copy of *rhea* did not cause GSC loss, but it did significantly (one-way analysis of variance, *P*<0.0001) suppress the phenotype of *Madm* knockdown testes (*C587*^ts^>*Madm*^RNAi^; Supplementary Fig. 9). These results suggest that Madm normally prevents CySCs from outcompeting GSCs for niche occupancy via an integrin-mediated mechanism.

**Figure 4:**
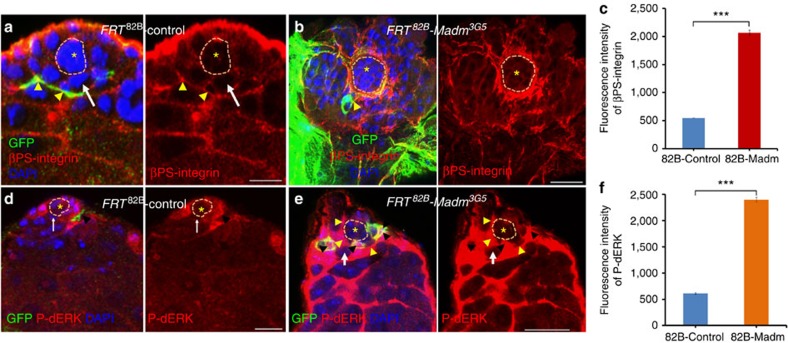
Madm regulates integrin and p-dERK expression non-cell autonomously. (**a**,**b**) GFP^+ve^ clones were generated in the testes of wild-type control (*FRT*^82B^*-PiM* (**a**, *n*=27) and *FRT*^82B^*-Madm*^3G5^ (**b**, *n*=32) clones at 3 days ACI. βPS-integrin expression is markedly increased in *Madm*^3G5^ testes at the CySCs–hub interface compared with wild-type control testes. The testes were immunostained with βPS-integrin (red), GFP (green) and DAPI (blue). GSCs are highlighted by white arrows. GFP-positive clones are highlighted by arrowhead (yellow, **a**,**b**). (**c**) A bar graph showing the quantitation of fluorescence intensity of βPS-integrin in *FRT*^82B^*-PiM* and *FRT*^82B^*-Madm*^3G5^ testes at the CySC–hub interface. (**d**,**e**) GFP-positive clones were generated in the testes of wild-type control (*FRT*^*82B*^*-PiM* (**d**, *n*=35) and *FRT*^82B^*-Madm*^3G5^ (**e**, *n*=37) clones at 3 days ACI. p-dERK expression is markedly increased in *FRT*^*82B*^*-Madm*^*3G5*^ testes in both GFP-positive cells and GFP^−ve^ cells compared with wild-type control testes. The testes were immunostained with p-dERK (red), GFP (green) and DAPI (blue). GFP-positive clones are highlighted by arrowhead (black, **d**,**e**) and GFP-positive cells are highlighted by arrowhead (yellow, **d**,**e**). GSCs are highlighted by white arrows. (**f**) A bar graph showing the quantitation of fluorescence intensity of p-dERK in *FRT*^82B^*-PiM* and *FRT*^82B^*-Madm*^3G5^ testes. Asterisks with orange dotted lines indicate hub cells. All values are mean±s.e.m. Statistical significance determined by Student's *t*-test, ***P<0.0001. Scale bar, 10 μm (**a**,**b**,**d**,**e**).

We also examined βPS-integrin expression in GFP positively marked CySC MARCM clones of wild-type or *Madm* flies (Fig. 4a,b). Surprisingly, we found that the βPS-integrin level was significantly (Student's *t*-test, *P*<0.0001) higher in both GFP-positive *Madm* mutant CySCs and GFP-negative wild-type CySCs in the *Madm* testes (Fig. 4b,c) than the level in the wild-type testes (Fig. 4a,c), particularly at the CySC–hub interface, indicating that *Madm* regulates integrin expression non-cell autonomously.

Similarly, we examined the expression of phosphorylated *Drosophila* ERK (p-dERK) in GFP positively marked CySC MARCM clones of wild-type or *Madm* testes (Fig. 4d,e). Again, we were surprised to find that the p-dERK level was significantly (Student's *t*-test, *P*<0.0001) higher in both GFP-positive *Madm* mutant CySCs and GFP-negative wild-type CySCs in the *Madm* testes (Fig. 4e,f) than the level in the wild-type testes (Fig. 4d,f), indicating that *Madm* regulates p-dERK expression non-cell autonomously. We further tested the genetic interaction between *Madm* and its components in the EGFR signal transduction pathway and found that reducing the dosage of several components in the EGFR pathway significantly (one-way analysis of variance, *P*<0.0001) suppressed the phenotype of the *Madm* knockdown testes (*C587*^ts^>*Madm*^RNAi^; Supplementary Fig. 9). These results suggest that the EGFR signal transduction pathway functions either downstream or in parallel to Madm in regulating the competition between CySCs and GSCs for niche occupancy.

### Mlf1 and Madm play opposite roles in stem cell competition

Madm and the oncogene Mlf1 have opposing functions in regulating M1 cell differentiation^45^. We examined the function of Mlf1 in regulating stem cell competition by examining the expression of Zfh-1 and Stat92E in *C587*^ts^>Mlf1 testes. In the wild-type testes, the nuclei of Zfh-1-positive CySCs are farther from the hub than the nuclei of GSCs (Supplementary Fig. 10a). In both *C587*^ts^>*Madm*^RNAi^ (Supplementary Fig. 10b) and *C587*^ts^>Mlf1 testes (Supplementary Fig. 10c), after shifting to the restrictive temperature, the Zfh-1-positive CySCs gradually moved into the niches and pushed GSCs out (Supplementary Fig. 10b,c). We also found that CySCs and hub cells had elevated levels of Stat92E in *C587*^ts^>Mlf1 testes compared with control testes (Supplementary Fig. 10d–f), similar to those in *C587*^ts^>*Madm*^RNAi^ testes (Supplementary Fig. 7b–e). These data together suggest that the oncogene Mlf1 and tumour suppressor Madm play opposite roles in regulating stem cell competition in the *Drosophila* testis.

### The Ras-Raf pathway regulates CySCs–GSCs competition

**Figure 5:**
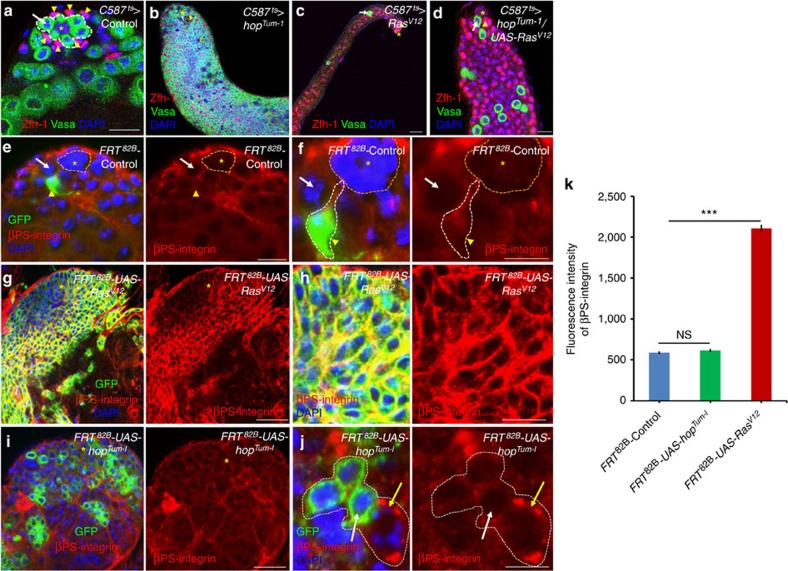
The Ras, rather than the JAK (Hop), regulates the competition between CySCs and GSCs for niche occupancy. (**a**–**d**) Confocal sections of the testes containing *C587*^ts^*>*Control (**a**, *n*=26), *C587*^ts^*>hop*^*Tum*−*l*^ (**b**, *n*=25), *C587*^ts^*>Ras*^*V12*^ (**c**, *n*=20), and *C587*^ts^*>hop*^*Tum*−*l*^*/Ras*^*V12*^ (**d**, *n*=27). The testes were cultured for 6 days at 29 °C before they were immunostained with Zfh-1 (red), Vasa (green) and DAPI (blue). GSCs are highlighted by white arrows and white dotted lines near hub cells (**a**–**d**). CySCs are highlighted by arrowhead (yellow, **a**–**d**). (**e**–**k**) GFP^+ve^ clones were generated in the testes of *FRT*^82B^*-PiM* (**e**,**f**), *FRT*^82B^*-UAS-Ras*^*V12*^ (**g**,**h**; *n*=45) and *FRT*^82B^*-UAS-hop*^*Tum*−*l*^ (**i**,**j**; *n*=37) flies using the MARCM technique and were stained at 6 days ACI with antibodies against GFP (green) and βPS-integrin (red). DAPI (blue) stained nuclei. In *FRT*^82B^*-UAS-Ras*^*V12*^ testes integrin is highly elevated in GFP^+ve^ CySC clones (**g**,**h**); however, in *FRT*^82B^*-UAS-hop*^*Tum*−*l*^ testes, integrin is weakly expressed (**i**,**j**). GSCs are highlighted by white arrows. (**k**) A bar graph showing the quantitation of fluorescence intensity of βPS-integrin in *FRT*^82B^*-PiM*, *FRT*^82B^*-UAS-Ras*^*V12*^ and *FRT*^82B^*-UAS-hop*^*Tum*−*l*^ testes. *indicate hub cells. All values are mean±s.e.m. Statistical significance determined by Student's *t*-test, ***P<0.0001. Scale bar, 10 μm (**a**–**j**).

The data described above indicate that both the JAK-STAT and EGFR signal transduction pathways may function downstream of Madm in regulating stem cell competition through integrin. However, *hop*^*Tum*−*l*^ (the activated JAK kinase) overexpression in CySCs (*C587*^ts^>*hop*^*Tum*−*l*^) did not produce the stem cell competition phenotype rather than GSC tumours^19^,^57^ (Fig. 5b, compare with Fig. 5a). To examine the function of the EGFR signal transduction in regulating the competition between CySCs and GSCs for niche occupancy, we expressed a constitutively activated form of Ras (*Ras*^*V12*^) and a gain-of-function mutant form of Raf (*Raf*^*gof*^) in somatic cells (*C587*^ts^>*Ras*^*V12*^, Fig. 5c and Supplementary Fig. 11a; and *C587*^ts^>*Raf*^*gof*^; Supplementary Fig. 11c). Elevation of EGFR signalling through *Ras*^*V12*^ or *Raf*^*gof*^ expression in somatic cells resulted in CySC overproliferation and CySCs outcompeting GSCs for niche occupancy (Fig. 5c, Supplementary Figs 11a,c), similar to what was seen in phenotypes of knocking down *Madm* in somatic cells (Figs 1c–e and 2b,c). We further expressed *hop*^*Tum*−*l*^ with *Ras*^*V12*^ (*C587*^ts^>*hop*^*Tum*−*l*^*/Ras*^*V12*^; Fig. 5d and Supplementary Fig. 11b) or *hop*^*Tum*−*l*^ with *Raf*^*gof*^ (*C587*^ts^>*hop*^*Tum*−*l*^*/Raf*^*gof*^; Supplementary Fig. 11d) in somatic cells and found that the testes expressing the double transgenes had phenotypes similar to the testes expressing *Ras*^*V12*^ or *Raf*^*gof*^ alone. These results suggest that the EGFR/Ras/Raf/ERK pathway functions either downstream or in parallel of the JAK signal transduction pathway in regulating the ability of CySCs to outcompete GSCs for niche occupancy. We also examined the expression of βPS-integrin in the testes with GFP-positive marked CySC MARCM clones of control (Fig. 5e,f), *Ras*^*V12*^ (Fig. 5g,h) and *hop*^*Tum*−*l*^ (Fig. 5i,j) or in *C587*^ts^>*hop*^*Tum*−*l*^ (Supplementary Fig. 11e) and *C587*^ts^>*Ras*^*V12*^ (Supplementary Fig. 11f) testes. We found that only *Ras*^*V12*^ induced βPS-integrin expression in these experiments (Fig. 5h,k), which was consistent with the above functions of *hop*^*Tum*−*l*^ and *Ras*^*V12*^ in regulating competition between CySCs and GSCs for niche occupancy.

These data together suggest that the EGFR/Ras/Raf/ERK pathway may function downstream of the JAK-STAT signal transduction pathway in regulating the competition between CySCs and GSCs for niche occupancy by controlling integrin expression.

### The JAK pathway regulates nuclear translocation of Madm

**Figure 6:**
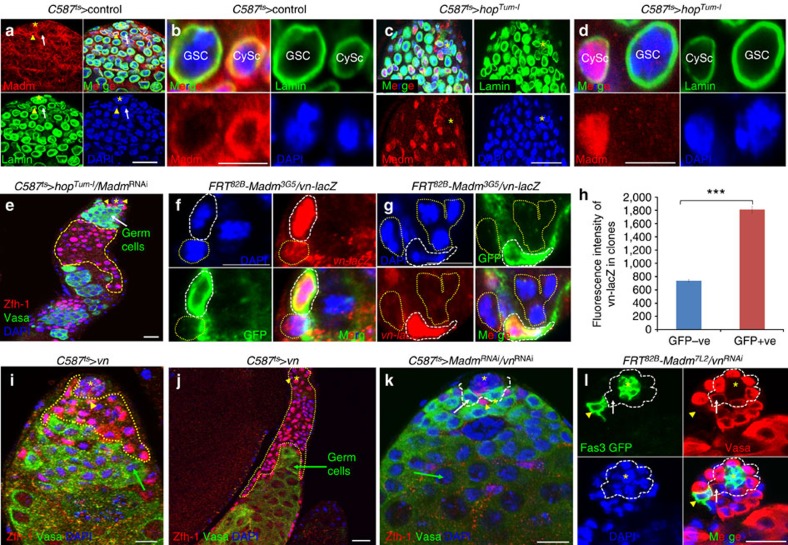
JAK signaling regulates the nuclear translocation of Madm, and Madm negatively regulates the EGFR/Ras/ERK signaling pathway by repressing *vn* expression. (**a**–**d**) The testes of *C587*^ts^*>* Control (6 days, **a**,**b**; *n*=15), *C587*^ts^*>hop*^*Tum*−*l*^ (6 days; **c**,**d**; *n*=32) were immunostained with the Madm (red), Lamin (red) and DAPI (blue). *indicate hub cells (**a**,**c**). GSC is highlighted by white arrows and CySCs are highlighted by yellow arrowhead (**a**,**c**). (**e**) The testes of *C587*^ts^*>hop*^*Tum*−*l*^*/Madm*^RNAi^ (6 days, *n*=25). Testes were cultured for 7 days at 29 °C before they were immunostained with Zfh-1 (red), Vasa (green) and DAPI (blue). (**f**,**g**) The testes of *FRT*^82B^*-Madm*^*3G5*^*/vn-lacZ* (*n*=33) were immunostained with β-galactosidase (*vn*-*lacZ*-red), GFP (green) and DAPI (blue). *vn* expression is markedly increased in GFP-positive *Madm* mutant CySCs (white dotted lines) compared with GFP-negative CySCs (yellow dotted lines). (**h**) A bar graph showing the quantitation of fluorescence intensity of *vn-LacZ* in *FRT*^82B^*-Madm* mutant clones. (**i**–**k**). Confocal sections of the testes containing *C587*^ts^*>vn* (**i** (70%) and **j** (30%); *n*=32); and *C587*^ts^*>Madm*^RNAi^*/vn*^RNAi^ (**k**, *n*=30). The testes were cultured for 7 days at 29 °C before they were immunostained with Zfh-1 (red), Vasa (green) and DAPI (blue). (**l**) The testes of *FRT*^82B^*-Madm*^*7L2*^*/vn*^*RNAi*^ (*n*=22) were immunostained 4 days ACI with GFP (green, yellow arrowhead), Fas3 (green, hub cells), Vasa (red) and DAPI (blue). *vn* knockdown in GFP-marked MARCM CySC clones of *Madm* mutant significantly (Student's *t*-test, *P*<0.0001) suppress the phenotypes associated with *Madm* knockdown. Green arrows indicate differentiated germ cells (**e**,**i**,**j**). Yellow arrowhead indicate CySCs (**e**,**i**–**l**). GSC are highlighted by white arrows (white dotted lines, **k**,**l**). Yellow dotted lines indicate Zfh-1-positive cells (**e**,**i**,**j**). *indicate hub cells. All values are mean±s.e.m. Statistical significance determined by Student's *t*-test, ***P<0.0001. Scale bar, 10 μm (**a**–**h**).

To explore the relationship between Madm and the JAK-STAT signal transduction pathway, we compared Madm protein expression in wild-type (Fig. 6a,b) and *C587*^ts^>*hop*^*Tum*−*l*^ testes (compare Fig. 6c,d with Fig. 6a,b), and found that most Madm translocated into the nuclei in the *C587*^ts^>*hop*^Tum−l^ flies (Fig. 6c,d), while the Madm protein was mostly localized to the cytoplasm in wild-type flies (Fig. 6a,b). We further found that expressing *C587*^ts^>*hop*^*Tum*−*l*^/*Madm*^RNAi^ produced a phenotype similar to expressing *C587*^ts^>*Madm*^RNAi^ (Fig. 6e), suggesting that *hop*^*Tum*−*l*^ regulates stem cell competition through Madm.

We also examined Madm expression in *C587*^ts^>*Stat92E*^AC^ (a constitutively activated version of Stat92E) and *C587*^ts^>*Socs36E*^RNAi^ testes, and we did not find increasing nuclear translocation of Madm (Supplementary Fig. 12a), suggesting that Stat92E and Socs36E do not regulate Madm nuclear translocation. We further generated *Socs36E*^RNAi^ MARCM clones (Supplementary Fig. 12b–d) and found that only GFP-positive CySCs were moved to the tip of the testes, and outcompete GSCs for niche occupancy. These data together suggest that only activated JAK (*hop*^*Tum*−*l*^) regulates Madm nuclear translocation, and Socs36E may function in the EGFR signal transduction pathway and regulate stem cell competition cell autonomously.

### Madm represses the expression of EGFR ligand *vein* in CySCs

**Figure 7:**
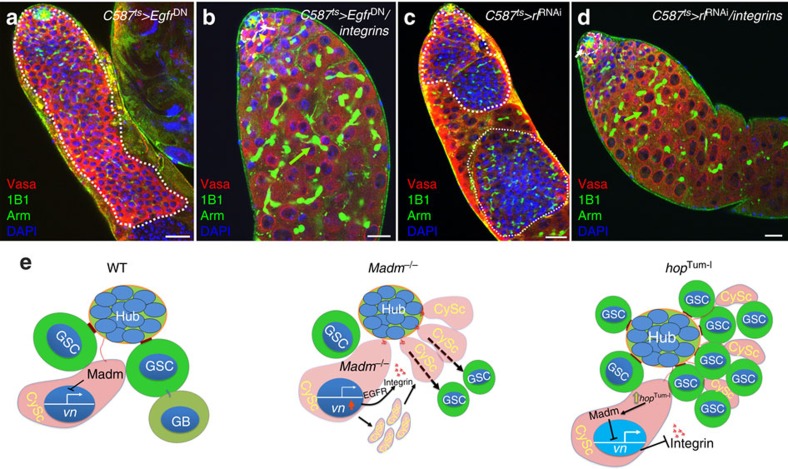
The overexpression of integrin rescued the GSC tumour phenotypes associated with the loss of EGFR-ERK signaling and overexpression of *Zfh-1.* (**a**–**d**) GSCs in the testes of *C587*^ts^*>Egfr*^DN^ (**a**, *n*=22), *C587*^ts^*>Egrf*^DN^*/PS1βPS (integrins)* (**b**, *n*=32), *C587*^ts^*>rl*^RNAi^(**c**, *n*=30) and *C587*^ts^*>rl*^RNAi^*/PS1βPS (integrins)* (**d**, *n*=35). The testes were cultured for 7 days at 29 °C before they were immunostained with Vasa (red), 1B1 and Arm (green) and DAPI (blue). *indicate hub cells. GSCs are highlighted by white arrows (**b**,**d**). CySCs are highlighted by arrowhead (yellow, **b**,**d**). White dotted lines in a and c representing GSC tumour phenotype. (**e**) A model of how Madm regulates GSC and CySC competition in *Drosophila* testis. Details are described in the text. Scale bar, 10 μm (**a**–**d**).

We further investigated the relationship between Madm and the EGFR signal transduction pathway. We demonstrated that knockdown of *Madm* non-cell autonomously induced p-dERK expression (Fig. 4d–f), and our data also suggest that the EGFR signal transduction pathway may function downstream of Madm and the JAK-STAT signal transduction pathway in regulating stem cell competition. This information indicates that Madm may regulate stem cell competition by repressing EGFR ligand expression in the testis. *Spitz (Spi)* and *Vein (Vn)* are two major ligands of EGFR^58^. We examined their gene expressions in the testes using their respective LacZ reporters. Consistent with a previous finding^59^, we found that *spi* is expressed in germ cells (Supplementary Fig. 12e); *vn* was mainly expressed in somatic cells, including CySCs (Supplementary Fig. 12f). To further investigate the relationship between Madm mutation and *vn* expression, we generated GFP-marked MARCM CySC clones of *Madm* mutant with *vn-lacZ* (Fig. 6f–h) and found that *vn* expression was markedly increased in GFP-positive *Madm* mutant CySCs compared with that in GFP-negative wild-type CySCs (Fig. 6f–h). We also generated the flip-out clones in the testes of *hs-flp; Ay>Gal4/UAS-GFP+UAS-Madm*^RNAi^*+vn-LacZ* flies and again found that *vn* expression was markedly increased in GFP-positive *Madm*^RNAi^ CySCs compared with that in GFP-negative wild-type CySCs (Supplementary Fig. 12g,h). These data together suggest that Madm represses *vn* expression in CySCs. We further found that *vn* overexpression promoted the CySCs' ability to outcompete GSCs for niche occupancy (similar to the phenotype of *Madm* knockdown; Fig. 6i,j). Knockdown of *vn* alone in CySCs results in GSC tumour phenotypes (Supplementary Fig. 12i), similar to those of knockdown of *Egfr* (Fig. 7a). Co-knockdown of *vn* and *Madm* in CySCs suppressed the phenotype of *Madm* knockdown (Fig. 6k) and expression of *vn*^RNAi^ in *Madm* mutant MARCM CySC clones restored the testes to the phenotypes of wild-type testes (Fig. 6l, compare Fig. 6l with Supplementary Fig. 6d–g). These data demonstrated that *vn* is the major downstream target of *Madm*.

We also found that the expression of a constitutively activated form of EGFR (*C587*^ts^>*λtop*; Supplementary Fig. 13a,b) could promote the ability of CySCs to outcompete GSCs for niche occupancy and stimulate Stat92E expression in CySCs (Supplementary Fig. 13b,c). These data together suggest that in such a model knocking down *Madm* may first result in *vn* overexpression, which further activates the EGFR signal transduction pathway non-cell autonomously to activate Stat92E and induce integrin expression in CySCs, so that CySCs outcompete GSCs for niche occupancy.

### Integrin overexpression can rescue the GSC tumour phenotypes

Our data described above demonstrated that the activated EGFR/Ras signal transduction pathway regulates integrin expression, and that integrin plays a pivotal role in controlling the competition between CySCs and GSCs for niche occupancy. Loss-of-function mutations in the EGFR signal transduction pathway^17^,^27^ or overexpression of *zfh-1* and *hop*^*Tum*−*l*^ resulted in GSC tumour phenotypes^19^. We examined the function of integrin in regulating GSC tumour phenotypes associated with expressing a dominant-negative form of EGFR (*Egfr*^DN^; Fig. 7a), *dERK* RNAi (*rl*^RNAi^; Fig. 7c), or *zfh-1* (Supplementary Fig. 13d) in somatic cells. We found that integrin overexpression could significantly (Student's *t*-test, *P*<0.0001) rescue the GSC tumour phenotypes associated with expressing a dominant-negative form of EGFR (*EGFR*^DN^; Fig. 7b), *rl*^RNAi^ (Fig. 7d), *zfh-1* (Supplementary Fig. 13e) and *hop*^*Tum*−*l*^ (Supplementary Fig. 14a) in somatic cells. These data suggest that the loss of EGFR-ERK signalling and overexpression of *zfh-1* and *hop*^*Tum*−*l*^ may downregulate integrin expression in CySCs, which is important for allowing GSCs to outcompete CySCs for niche occupancy and in promoting GSC tumour formation.

We further examined *vn* expression in the testes that overexpressed *hop*^*Tum*−*l*^ (*C587*^ts^>*hop*^*Tum*−*l*^/*vn-lacZ*) and found that *vn* expression was markedly suppressed (Supplementary Fig. 14b in comparison with Supplementary Fig. 12f). These data together suggest that expressing the activated JAK kinase (*hop*^*Tum*−*l*^) promotes Madm nuclear translocation, which further suppresses *vn* and integrin expression in CySCs that allows GSCs to outcompete CySCs for niche occupancy and promotes GSC tumour formation.

## Discussion

In this study, we have shown that the novel tumour suppressor Madm regulates the competition between GSCs and CySCs for niche occupancy in the adult *Drosophila* testis. We found that Madm is specifically expressed in CySCs and knockdown of *Madm* results in the overexpression of the EGF receptor ligand *vn*, which further activates EGF receptor signalling and integrin expression non-cell autonomously in CySCs to promote their overproliferation and ability to outcompete GSCs for niche occupancy. Conversely, expressing a constitutively activated form of *Drosophila* JAK kinase (*hop*^*Tum*−*l*^) promotes Madm nuclear translocation and suppresses *vn* and integrin expression in CySCs, allowing GSCs to outcompete CySCs in niche occupancy and promoting GSC tumour formation (Fig. 7e and Supplementary Fig. 14c). The loss of EGFR-ERK signalling or overexpression of *zfh-1* and *chinmo* in CySCs also resulted in GSC tumour formation^17^,^19^,^27^,^57^. We found that overexpressing integrin can significantly (Student's *t*-test, *P*<0.0001) rescue the GSC tumour phenotypes associated with the loss of EGFR-ERK signalling and overexpression of *zfh-1* and *hop*^*Tum*−*l*^. These results together suggest that the relative integrin level in CySCs determines whether GSCs or CySCs occupy the niche, while the tumour suppressor, and signal transduction pathways regulate stem cell competition and stem cell tumour formation by modulating integrin expression.

In human and mouse hematopoietic stem cells (HSCs), breast and other cancer systems, integrins play important roles in regulating stemness, stem cell competition and cancer stem cell formation^60^,^61^,^62^. It was recently reported that the reduced expression of CD18 (a leukocyte integrin) leads to a cell-autonomous *in vivo* expansion of early quiescent short-term and long-term HSCs in mouse bone marrow^61^. Remarkably, the CD18-low HSCs are more competitive than their wild-type counterparts in the bone marrow competition assays of recipient mice. Therefore, low integrins in this case make HSCs more competitive. In many other cases, the opposite seems to be true. High expression of the α6 integrin subunit (CD49f) is a biomarker for breast and other cancer stem cells^60^. A recent publication further demonstrated that integrin α_v_β_3_ serves as a marker/driver of carcinoma stemness that is highly resistant to receptor tyrosine kinase inhibitors such as erlotinib^62^. The integrin α_v_β_3_ regulates tumour initiation, anchorage independence, self-renewal and erlotinib resistance by activating the KRAS-RalB-NF-κB pathway. In this study, we further revealed the molecular details of how a tumour suppressor and the signal transduction pathways of JAK and EGFR coordinately regulate stem cell competition and stem cell tumour formation by manipulating integrin expression in one type of stem cells.

Stem cell competition generally involves three steps. The competitive stem cells first become more fit before they move and anchor to a special location (such as a niche) by expressing cell-adhesion molecules, and finally eliminate the outcompeted stem cells. In the mouse thymus, the ‘young' bone marrow-derived progenitors are more fit; they move to the thymus to gain survival advantages by contacting the survival factor IL-7 and increasing the expression of intracellular pro-survival protein Bcl2. The ‘old' thymus-resident progenitors have low survival advantages because they have low affinity to IL-7, express low levels of Bcl2, and are eliminated through apoptosis^4^. However, it is not clear what changes make the bone marrow-derived progenitors more fit and what molecules mediate the young progenitors' move to the thymus and cause them to remain there. In *p53*-deficient mice, the *p53*-low hematopoietic stem and progenitor cells are more competitive (have advantages in proliferation and long-term maintenance), may be located to a special microenvironment (niche), and also induce growth arrest and senescence-related gene expression non-cell autonomously in the outcompeted *p53*-high hematopoietic stem and progenitor cells by expressing an unknown secreting factor^1^,^8^. In the *Drosophila* ovary, the differentiation-defective *bam*- or *bgcn*-mutant GSCs are more competitive, and they invade the niche space of neighbouring wild-type GSCs by upregulating the adhesion molecule E-cadherin. The detached wild-type GSCs from the niche will go through differentiation^3^. In this case, the self-renewal-promoting BMP signal is not required in stem cell competition. However, in another study, it was found that the GSCs expressing high levels of *Drosophila Myc* (dMyc) were more competitive than the dMyc-low GSCs^5^. The niche-provided self-renewal factor BMP/DPP in metabolically active dMyc-high GSCs is required for stem cell competition.

In the *Drosophila* testis, we demonstrated that the tumour suppressor *Madm-*deficient CySCs are more competitive. The *Madm-*deficient CySCs overexpress the EGF receptor ligand *vn*, which secretes to the intercellular fluid and activates EGF receptor signalling non-cell autonomously in CySCs to stimulate CySC proliferation and increase integrin expression. The elevated integrin gives CySCs the advantage of outcompeting GSCs for niche occupancy. The niche signal Upd may activate JAK-STAT signalling in CySCs to further enhance fitness and maintenance of CySCs. The outcompeted GSCs will leave the niche and become differentiated. Conversely, promoting Madm nuclear translocation by expressing the *hop*^*Tum*−*l*^ or knocking down EGFR signalling will suppress integrin expression in CySCs, which will give GSCs the advantage of outcompeting CySCs for niche occupancy and will result in GSC tumour formation.

*p53* and *Madm* share some similarities in regulating stem cell competition. They both regulate stem cell competition non-cell autonomously. In mice, the *p53*-deficient HSCs may secrete an unknown factor that non-cell autonomously promotes proliferation and long-term maintenance in the *p53*-low cells and, at the same time, induces growth arrest and senescence-related gene expression in the *p53*-high cells^1^,^8^. In *Drosophila*, our study demonstrated that *Madm*-deficient CySCs induce the expression of the EGFR ligand *vn*, which further activates EGF receptor signalling and integrin expression non-cell autonomously to promote CySC proliferation and the ability of CySCs to outcompete GSCs for niche occupancy. Furthermore, unlike what often happens in other cell competitions^63^, the outcompeted ‘loser' stem cells were not eliminated through cell death. In the *p53* case, the loser HSCs became senescent; in the *Madm* case, the loser GSCs became differentiated. Altogether, these data suggest that stem cell (or cancer stem cell) competition may be a fundamental mechanism of tumour formation. The knowledge gained from studying tumour suppressor-mediated stem cell competition will help to understand the unique molecular basis of tumour initiation and may be used to develop novel drug targets for advancing cancer therapy.

## Methods

### *Drosophila* stocks and cultures

Oregon R was used as the wild-type strain. The other fly stocks used in this study are either described in FlyBase or as otherwise specified. *Nos-Gal4* (*nanosGal4VP16*) (ref. 64), *Act-Gal4*, *tub-GAL80*^*ts*^, *Act5C-GAL4.UAS-GFP*, *UAS-GFP.nls*, *UAS-mCD8-GFP hs-Flp*, *SM6*, *hs-Flp*, and *tub-Gal4 FRT82B-tub-Gal80*, *UAS-Ras*^*V12*^, *UAS-Raf*^*gof*^*, Egfr*^*F24*^, *UAS-Mlf1*, *Egfr*^*DN*^, *UAS-Zfh-1*, *spi-lacZ* (BL10462), *vn-lacZ* (BL11749), *UAS-GFP*^RNAi^ and *Madm*^RNAi^ lines*-BL31644 (Madm*^RNAi−1^), BL41599, BL42529 were obtained from the Bloomington stock centre; *rl*^RNAi^ (v35641), *Madm*^RNAi−2^ (v27346) and *Socs36*^RNAi^ (v51821) were obtained from Vienna *Drosophila* Resource Centre (VDRC); *vn*^RNAi^ (10491R-2) was obtained from the National Institute of Genetics (NIG), Japan; *Drk*^TZ160^ and *Ras*^*c40b*^ are our lab stocks; *rl*^*698*^ is from Norbert Perrimon; *upd-Gal4* and *c587-Gal4* were provided by Ting Xie; *UAS-hop*^*Tum*−*l*^ was provided by Erika Bach and Soichi Tanda*; UAS-vn* was provided by Jocelyn Donaldson and Amanda Simcox; *UAS-PS1 βPS* (*integrins*) was provided by Kendal Broadie; *rhea*^*6*−*66*^ and *rhea*^*13*−*8*^ were provided by Amin Ghabrial; *UAS-λtop* was provided by Trudi Schupbach; FRT^82B^-*Madm*^*3G5*^ and FRT^82B^-*Madm*^*7L2*^ were provided by Hugo Stocker.

Flies were raised on standard fly food at 25 °C and at 65% humidity, unless otherwise indicated. Genes and mutations that are not described in the text can be found at Flybase (http://flybase.bio.indiana.edu).

### RNAi-mediated gene depletion

Male *UAS-RNAi* transgene flies were crossed with female virgins of genotype *Nos-Gal4*, *upd-Gal4*, *c587-Gal4; tub-Gal80*^ts^ (*c587*^ts^) or *Act-Gal4; tub-Gal80*^ts^ (*Act*^ts^). The flies were cultured at 18 °C. Three- to 5-day-old adult flies with the appropriate genotype were transferred to new vials at 29 °C for 1 to 15 days before dissection. The sequences used for VDRC knockdown strains are available for each line at https://stockcenter.vdrc.at, and sequences for Bloomington knockdown strains are available for each line at http://flystocks.bio.indiana.edu. These sequences are also shown in Supplementary Note 1.

### Generating mutant GSCs clones

Clones of mutant GSCs were generated as previously described^31^. To generate *Madm* mutant GSC clones, *FRT*^82B^/*+* and *FRT*^82B^-*Madm*^*3G5*^/*Cyo* or *FRT*^82B^-*Madm*^*7L2*^/*Cyo* virgin females were mated with males of genotype *FRT*^82B^-*arm-lacZ*/*Cyo*; *SM6, hs-flp/+.* Genotypes used to induce mutant clones are provided in Supplementary Note 1. One- or 2-day-old adult males carrying an *arm-lacZ* transgene in *trans* to the mutant-bearing chromosome were heat-shocked four times at 37 °C for 1 h, at intervals of 8–12 h. The males were transferred to fresh food every day at 25 °C. The testes were removed 1, 2, 4, 7 and 12 days after the first heat-shock treatment and processed for antibody staining.

### MARCM clonal analysis

Clones of GFP-marked mutant CySCs for control (*FRT*^82B^*-PiM/*+); and mutants (*FRT*^82B^*-PiM/+; FRT*^82B^-*UAS*-*hop*^*Tum*−*l*^*/+; FRT*^82B^-*UAS***-***Ras*^*V12*^*/+; UAS***-***Socs36E*^RNAi^*/+; FRT*^82B^*-Madm*^*3G5*^*; FRT*^82B^*-Madm*^*7L2*^) were generated using the MARCM system^54^. Detail of genotypes used to induce MARCM clones are provided in Supplementary Note 1. Three- or 4-day-old adult female flies were heat-shocked twice at 37 °C for 45 min, with intervals of 8–12 h. The flies were transferred to fresh food daily after the final heat shock. The testes were removed at 1, 2 or 7 days after the first heat-shock treatment and processed for antibody staining.

### Immunofluorescence staining and microscopy

Immunofluorescence staining was performed as previously described with some modifications^31^. Briefly, the testes were dissected in 1 × PBS, transferred to 4% formaldehyde in 1 × PBS and fixed for 30 min. The testes were then washed in PBST (PBS containing 0.1% Triton X-100) for 3 times for 10 min each time and blocked with 2% goat serum in PBST for 1 h. Samples were incubated with primary antibody in 1 × PBST at 4 °C overnight. Samples were washed for three times, 10 min each time in 1 × PBST, then incubated with secondary antibody in 1 × PBST at room temperature for 2 h, washed as previously described, and mounted in VECTASHIELD with DAPI (Vector Labs).

The following antisera were used: rabbit polyclonal anti-Vasa antibody (1:1,000; a gift from Ruth Lehmann), rabbit polyclonal anti-β-Gal antibody (1:1,000; Cappel), mouse monoclonal anti-β-Gal antibody (1:100; Invitrogen), mouse monoclonal anti-Hts antibody 1B1 (1:20; DSHB); rabbit anti-Madm (1: 500; a gift from Hugo Stocker), rabbit polyclonal anti-GFP antibody (1:200; Invitrogen), mouse monoclonal anti-GFP antibody (1:100; Invitrogen), rabbit polyclonal anti-phosphorylated histone H3 (pH3) antibody (1:1,000; Upstate), guinea pig polyclonal anti-Zfh-1 (1:2,000; a gift from James Skeath), rabbit polyclonal anti-Stat92E (1:500; ref. 55), mouse anti-βPS-integrin (1:20; DSHB), rabbit anti-p-dERK (1:200; Cell Signalling), rat anti-bromodeoxyuridine (1:50; Serotec), mouse anti-Fas3, rat anti-DE-cadherin and mouse anti-armadillo (Arm) N7A1 (1:20; DSHB). Secondary antibodies were goat anti-mouse, goat anti-guinea pig and goat anti-rabbit IgG conjugated to Alexa 488 or Alexa 568 (1:400; Invitrogen). Apoptosis was detected via TUNEL with the ApopTag Red *In Situ* Apoptosis Detection Kit (Chemicon International) according to the manufacturer's instructions. DAPI (Invitrogen) and VECTASHIELD with DAPI (Vector Labs) were used to stain DNA. Confocal images were obtained by using a Zeiss LSM510 system, and were processed with Adobe Photoshop CS6.

### Bromodeoxyuridine Labelling

We followed the protocol described by Brawley and Matunis^10^. In brief, male flies were starved for 8–10 h, and then fed 100 mM bromodeoxyuridine (Chem-Implex International) in yeast and sucrose paste with added green food colour to monitor feeding for 3 h at 29 °C. Testes were dissected and fixed immediately or allowed to recover for 2–4 days at 18 °C before dissection. Testes were fixed in 4% paraformaldehyde (in 1 × PBS) for 30 min at room temperature, then washed 3 times with 1 × PBX, and rinsed in 1 × PBS. Testes were then incubated with DNaseI (Promega) for 30 min at 37 °C before immunostaining^31^.

### Quantification and statistical analysis

The number of GSCs/testis and CySCs/testis was counted using serial confocal reconstructions of the entire testis apex^2^. Vasa-positive cells (with spherical spectrosomes) contacting the hub were scored as GSCs. Zfh-1-positive cells contacting the hub were scored as CySCs as described by Issigonis *et al.*^2^, and Leatherman and Dinardo^19^. Statistical analyses of GSC and CySC numbers (mean±s.e.m.) were performed using GraphPad Prism program. Sample sizes (*n*) reported reflect the individual testis number. All experiments were performed in triplicates. *P* values were obtained between two groups using the Student's *t*-test or between more than two groups by analysis of variance. For all statistical analysis, differences were considered to be statistically significant at values of *P*<0.05. To quantify the strength of fluorescence of Stat92E, βPS-integrin, p-DERK and *vn-lacZ,* all images were taken with the same confocal settings. For quantification, mean intensities of several individual cells attaching directly to hub cells in individual testis were measured. The fluorescence intensity was measured using an LSM5 image Browser (Zeiss).

## Additional information

**How to cite this article**: Singh, S. R. *et al.* The novel tumour suppressor Madm regulates stem cell competition in the Drosophila testis. *Nat. Commun.* 7:10473 doi: 10.1038/ncomms10473 (2016).

## Supplementary Material

Supplementary Information Supplementary Figures 1-14 and Supplementary Note 1
