# Supplementary material for: The novel tumour suppressor Madm regulates stem cell competition in the Drosophila testis
Source: Nat Commun. 2016 Jan 21;7:10473. doi: 10.1038/ncomms10473 (PMC4736159; doi:10.1038/ncomms10473)
Supplement: Supplementary Information — Supplementary Figures 1-14 and Supplementary Note 1 [file ncomms10473-s1.pdf]

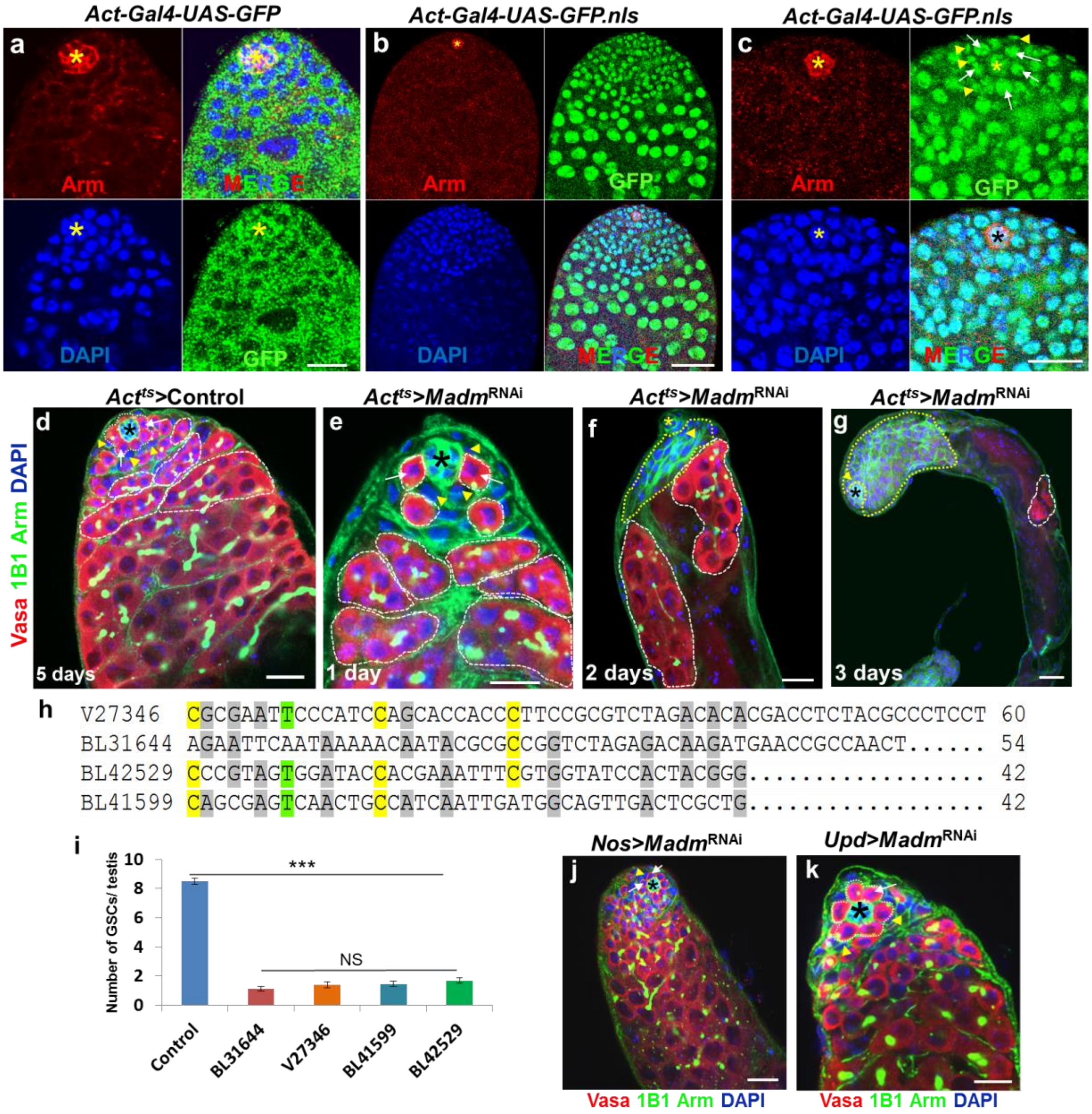

Supplementary Figure 1 | Madm is not required in GSCs and hub cells. (**a,b**) Act-Gal4-UAS-GFP (**a**), Act-Gal4-UAS-GFP.nls (**b,c**) is ubiquitously expressed in the testes. The testes were immunostained with GFP (green), Arm (red) and DAPI (blue). **(d–g)** GSCs in the testes containing *Act*^{ts}>Control (**d**, *n*=20), *Act*^{ts}>*Madm*^{RNAi} (**e**, 1 day, *n*=20; **f**, 2 days, *n*=27; and **g**, 3 days, *n*=35). The testes were immunostained with Vasa (red), 1B1 and Arm (green) and DAPI (blue). White arrows indicate GSCs (**c-e**). Yellow arrowheads indicate CySCs (**c-g**). Germ cells are highlighted by white dotted lines (**d-g**) and CySCs are highlighted by yellow dotted lines (**f,g**). (**h**) Sequences used to generate transgenic *Madm*^{RNAi} lines (v27346, BL31644, BL42529 and BL41599). (**i**) Quantitation of number of GSCs/ testis in four *C587>Madm*^{RNAi} lines (5 days, BL31644, *n*=35; v27346, *n*=29; BL41599, *n*=32; BL42529, *n*=38). NS, not significant; ****P*<0.0001. (**j,k**) GSCs in the testes containing *Nos>Madm*^{RNAi} (**j**, 7 days, *n*=30) and *Upd>Madm*^{RNAi} (**k**, 7 days, *n*=27). The testes were immunostained with Vasa (red), 1B1 and Arm (green) and DAPI (blue). White arrows indicate GSCs (**j,k**). Yellow arrowheads indicate CySCs (**j,k**). Asterisks indicate hub cells. All values are mean ± s.e.m. Statistical significance determined by one-way analysis of variance, ****P*<0.0001. Scale bars (**a–g** and **j, k**)**:** 10 μm.

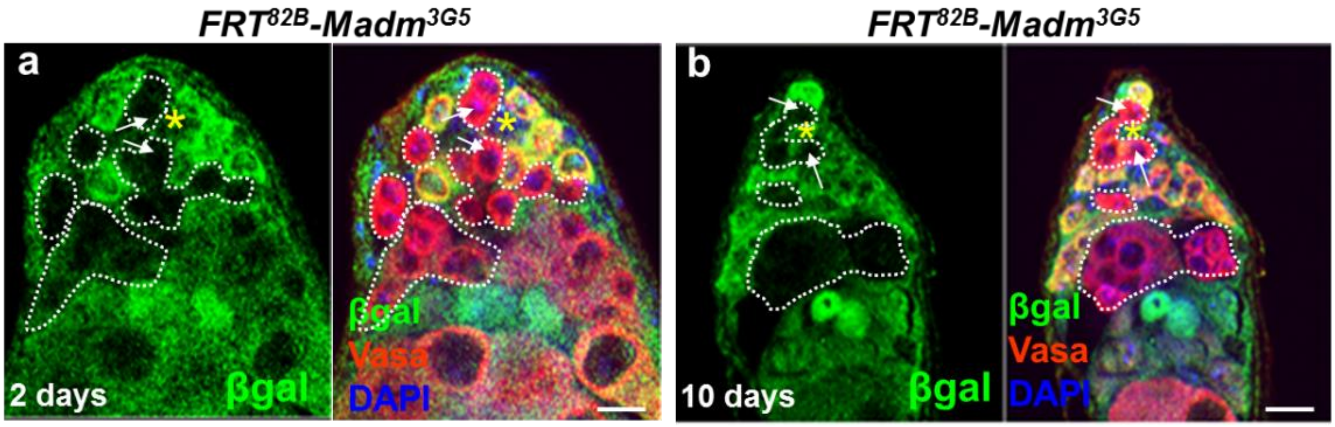

c

Madm **GSC clones**

Genotype	2d ACI	7d ACI	10d ACI
FRT^{82B}-$Madm^{7L2}$	0.85 (17/20)	0.83 (15/18)	0.73 (11/15)
FRT^{82B}-$Madm^{3G5}$	0.91 (20/22)	0.85 (17/20)	0.67 (12/18)

Supplementary Figure 2 | Madm is not required in germline stem cells. (**a,b**) The confocal sections through the apex of the testes containing *FRT*^{82B}-*Madm*^{3G5} clones at 2 days ACI (**a**) and 10 days ACI (**b**). The testes were immunostained with β-galactosidase (green), Vasa (red) and DAPI (blue). GSC clones are β-galactosidase negative. GSC clones are highlighted by white dotted lines. White arrows indicate GSCs. Asterisks indicate hub cells. **(c)** The Quantitative data of β-galactosidase–negative clones in *FRT*^{82B}-*Madm*^{3G5} and *FRT*^{82B}-*Madm*^{7L2} fly testes at 2, 7, and 10 days ACI. Scale bars (**a,b**)**:** 10 μm.

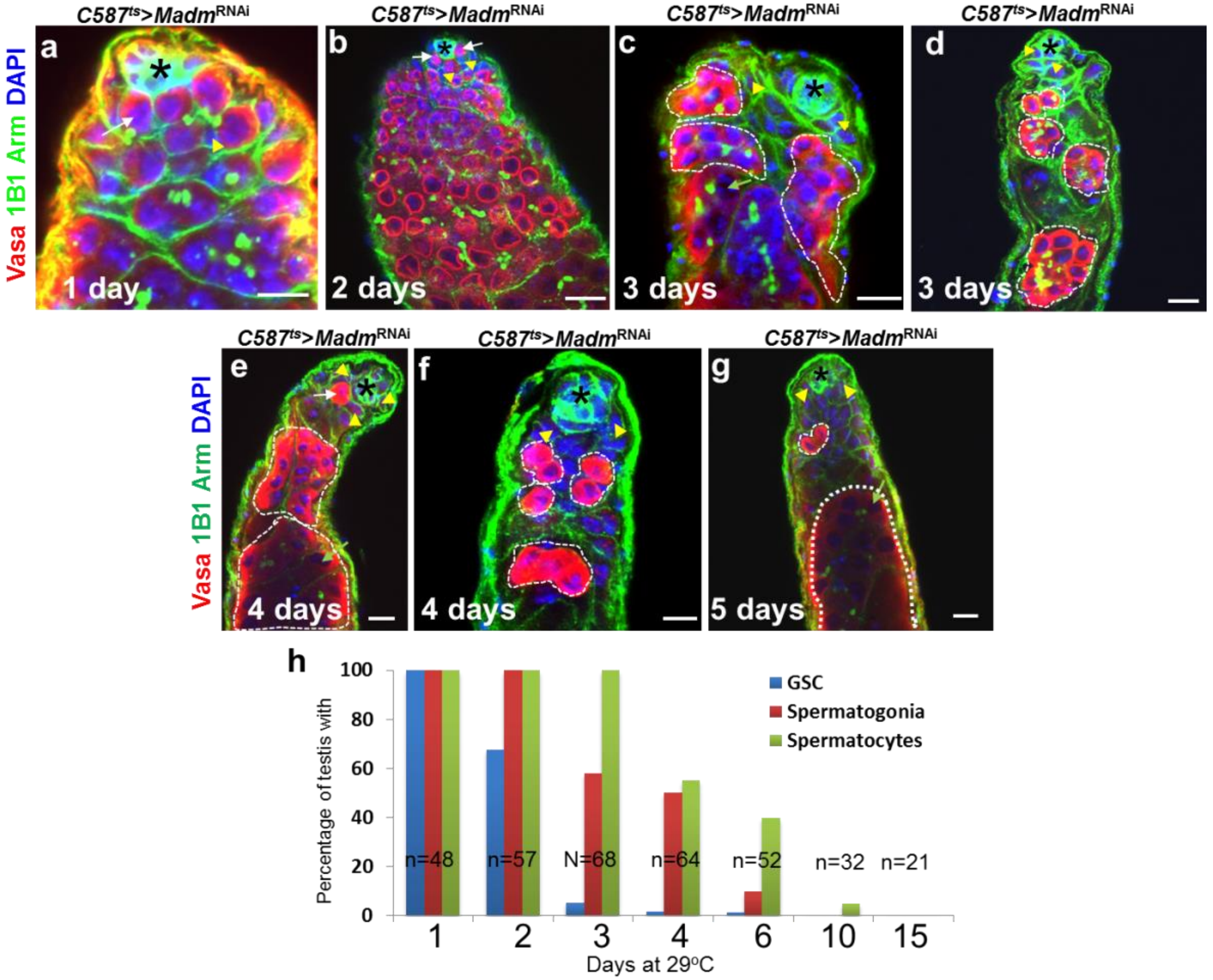

Supplementary Figure 3 | Madm functions in CySC to regulate GSC maintenance. (**a–g**) Representative examples of the confocal section of the testis apex from *C587*^{ts}> *Madm*^{RNAi} males after shifting from 18° to 29°C for 1 day (BL31644; **a**), 2 days (BL31644; **b**), 3 days (v27346; **c)** 3 days **(**BL41599; **d**), 4 days (BL31644; **e),** 4 days **(**BL42529; **f**) and 5 days (**g**) as adult flies. The testes were stained with Vasa (red), 1B1 and Arm (green) and DAPI (blue). White arrows indicate GSC, yellow arrowheads indicate CySCs, dotted lines indicate spermatogonial cells, and green arrows indicate spermatocytes. Asterisks indicate hub cells. (h) Quantitation of percentage of testis with GSCs, spermatogonia, and spermatocytes in *Madm*$^{RNAi-1}$ testes from 1 to 15 days at 29°C. Scale bars (**a–g**)**:** 10 μm.

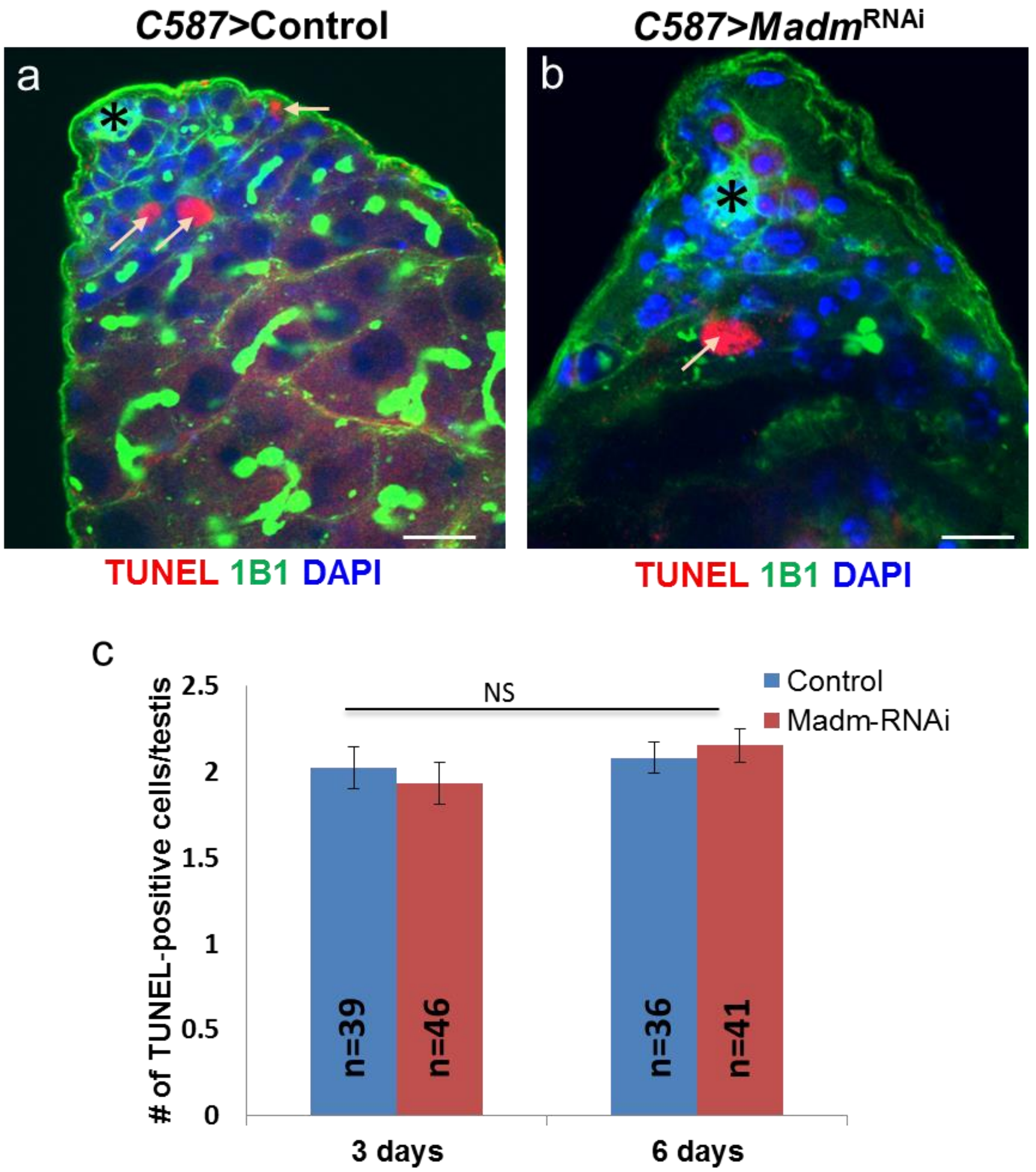

Supplementary Figure 4 | Loss of Madm resulted in GSC differentiation rather than cell death. (**a, b**) The confocal sections through the apex of the testes containing *C587*^{ts}>Control (**a**, 3 days, *n*=39), and *C587*^{ts}>*Madm*^{RNAi} (**b**, 3 days, *n*=46) stained with TUNEL labeling (red, orange arrows). The testes were immunostained with 1B1 and Arm (green) and DAPI (blue). Asterisks indicate hub cells. (**c**) Quantitation of number of TUNEL-positive cells/testis in *C587*^{ts}>Control, and *C587*^{ts}>*Madm*^{RNAi} from 3 and 6 days at 29°C. Error bars represent s.e.m. Statistical significance determined by Student's *t*-test, NS, not significant (P>0.05). Scale bars (**a,b**): 10 μm.

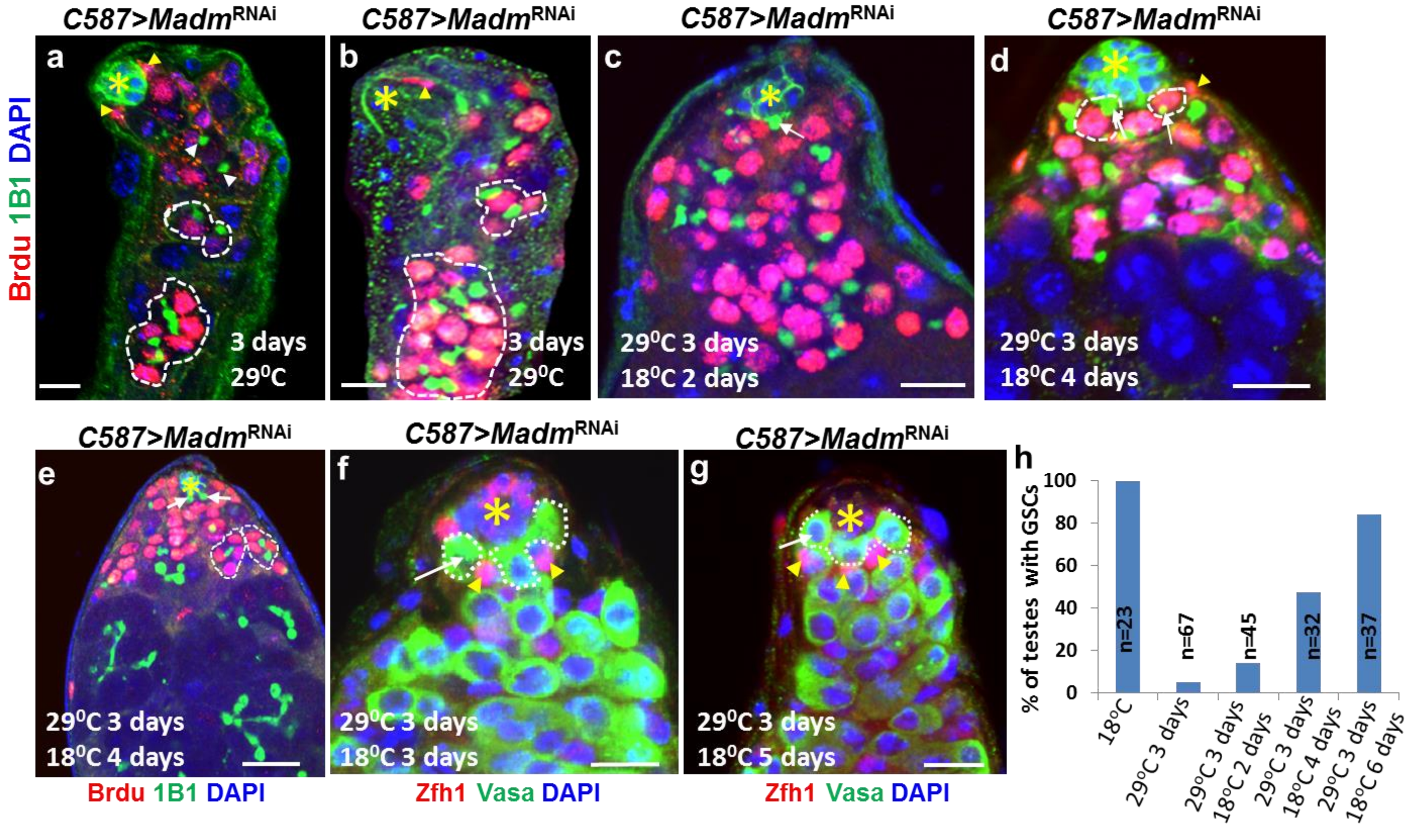

Supplementary Figure 5 | Spermatogonial dedifferentiation gives rise to new GSCs after restoring Madm. (**a–e**) The confocal sections through the apex of the testes containing *C587*^{ts}>*Madm*^{RNAi} shifted to 29°C for 3 days, then labeled by Brdu incorporation *in vivo* (**a, b**), 4 (rare), 8 and 16 cells spermatogonial cyst with branching fusome are found at 3 days far away from the hub (white dotted outlined). CySCs directly attached to the hub (arrowhead, yellow). Testis from *C587*^{ts}>*Madm*^{RNAi} shifted to 29°C for 3 days, then to 18°C for 2 days (**c**) and four days (**d, e**). GSCs with dot spectrosomes (arrow) contact the hub, 2 and 4 days after recovery at 18°C (**c, d**). Breakdown of spermatogonial cysts are detected at 2 days (**c**), and an accompanying partial cyst are labeled (white dotted outlined, **e**). (**f, g**) Testis from *C587*^{ts}>*Madm*^{RNAi} shifted to 29°C for 3 days, then to 18°C for 3 days (**f**) and 5 days (**g**). The testes were stained with Zfh-1 (red), Vasa (green) and DAPI (blue). White arrows indicate GSC, yellow arrowheads indicate CySCs. (**h**) Quantitation of percentages (%) of testes with GSCs shifted to 29°C for 3 days, then to 18°C for 3 to 6 days. Scale bars (**a–g**)**:** 10 μm.

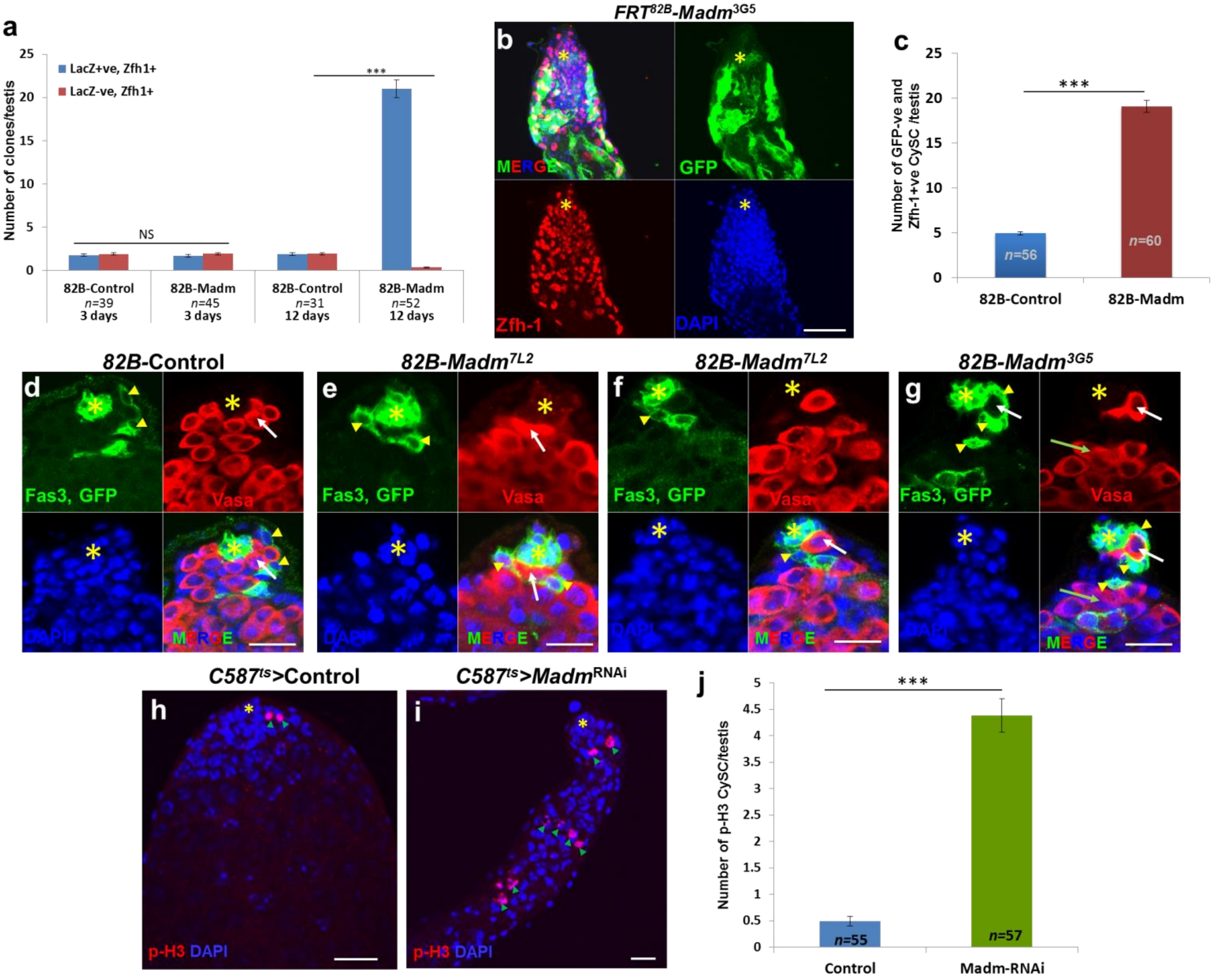

Supplementary Figure 6 | Madm functions in CySC non-cell-autonomously and regulates the competition between GSCs and CySCs and regulates CySC proliferation. (**a**) Quantitation of number of CySCs clones/testis with lacZ+ve, Zfh-1+ve versus lacZ-ve, Zfh1+ve cells in *FRT*^{82B}-Control and *FRT*^{82B}-*Madm*^{7L2}, 3 days and 12 days ACI. (**b**) GFP positive clones were generated in the testes of *FRT*^{82B}-*Madm*^{3G5} flies using the MARCM technique, and were stained at 7 days ACI with GFP (green), Zfh-1 (red) and DAPI (blue). (**c**) Quantitation of number of GFP-negative and Zfh-1+ve CySC/testis in *FRT*^{82B}-Control and *FRT*^{82B}-*Madm*^{3G5} at 7 days ACI. (**d–g**) GFP positive clones were generated in the testes of wild-type Control (*FRT*^{82B}-*PiM*, **d**; *n*=30), *FRT*^{82B}-*Madm*^{7L2} (**e,f;** *n*=25**)** or *FRT*^{82B}-*Madm*^{3G5} (**f,** *n*=27) flies using the MARCM technique, and were stained at 4 days ACI with GFP (green, yellow arrowhead), Fas3 (green, hub cells), Vasa (red) and DAPI (blue). GFP positive CySCs clones are highlighted by yellow arrowheads (**d-g**). White arrows indicate GSC and yellow asterisks indicate hub cells (**d-g**). (**h-j**) Mitotic cells are counted in the testes of *C587*^{ts}>Control (**h**), and *C587*^{ts}>*Madm*^{RNAi} (**i**). *C587*^{ts}>*Madm*^{RNAi} testes contain significantly increased number of pH3-positive cells compared to *C587*^{ts}>Control testes (**i**, pH3-positive cells per testis; 0.49±0.09, versus 4.38±0.32). Testes were cultured for 6 days at 29°C before they were immunostained with the pH3 antibody. Asterisks indicate hub cells. pH3 positive cells are highlighted by green arrowheads (**h,i**). All values are mean ± s.e.m. Statistical significance determined by Student's *t*-test, NS, not significant. ****P*<0.0001. Scale bars (**b, d-i**)**:** 10 μm.

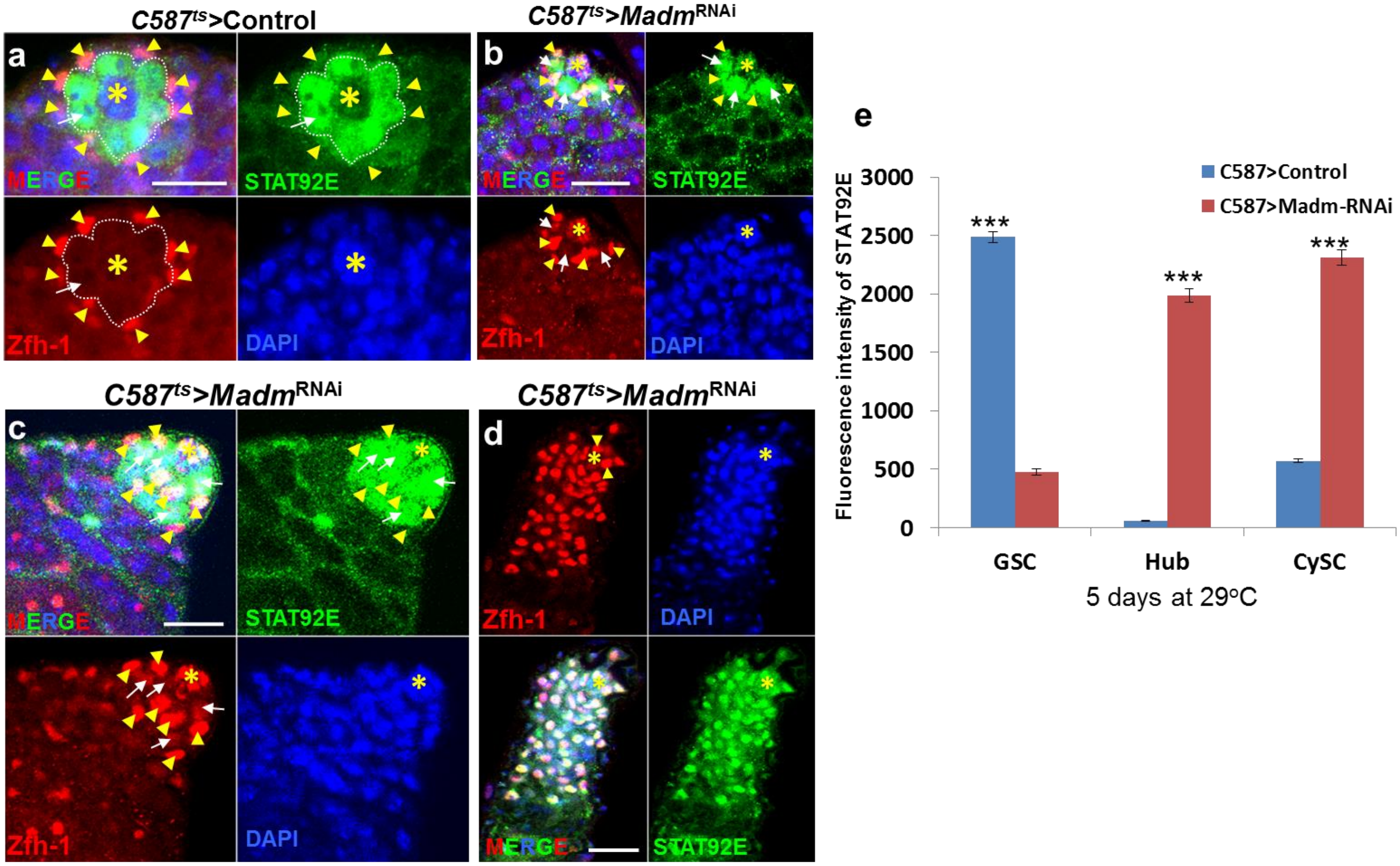

Supplementary Figure 7 | Madm attenuates JAK-STAT signaling in CySCs and hub cells to maintain the balance between GSCs and CySCs. (a–d) The confocal sections through the apex of the testes containing *C587*^{ts}>Control (**a**, 5 days at 29°C, *n*=20), and *C587*^{ts}>*Madm*^{RNAi} (**b**, 1 day at 29°C, *n*=22; **c**, 2 days at 29°C, *n*=17; **d**, 5 days at 29°C, *n*=25). The testes were immunostained with the antibodies against Stat92E (green) and Zfh-1 (red). In the control testis, Stat92E is enriched in GSCs compared to CySCs and hub cells (**a**). In *C587*^{ts}>*Madm*^{RNAi} testes, Stat92E levels increased dramatically in CySCs and hub cells compared to controls (**b-d**). White arrows indicate GSC. CySCs are highlighted by yellow arrowheads. Asterisks indicate hub cells. (**e**) A bar graph showing the quantitation of fluorescence intensity of Stat92E in *C587*^{ts}>Control and *C587*^{ts}>*Madm*^{RNAi} testes, 5 days at 29°C. All values are mean ± s.e.m. Statistical significance determined by Student's *t*-test, ***P<0.0001. Scale bars (**a–d**)**:** 10 µm.

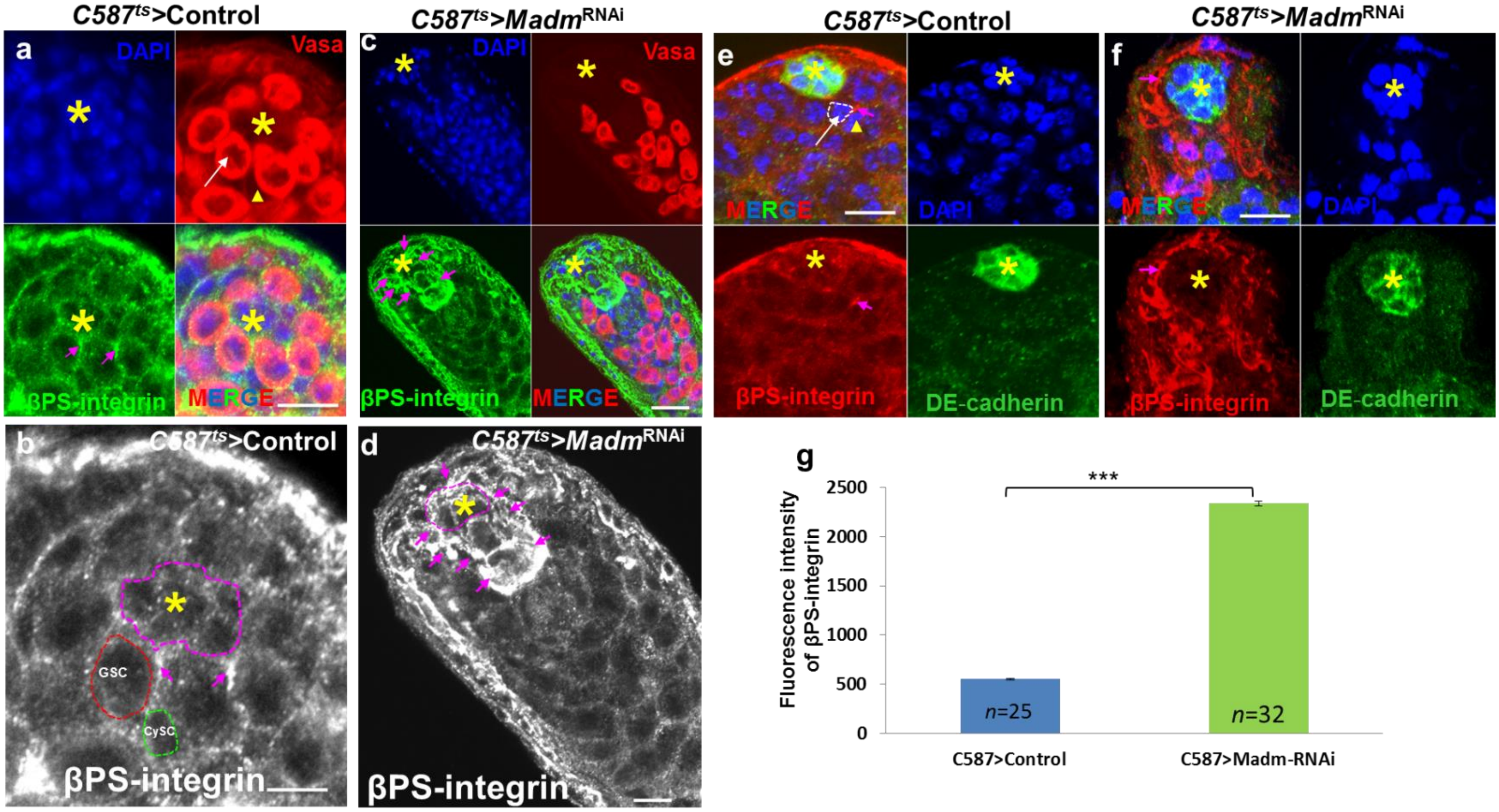

Supplementary Figure 8 | Integrin is elevated in *Madm*^{RNAi} testes at the CySCs–hub interface. (a–f) The testes containing *C587*^{ts}>Control (**a,b**, 5 days at 29°C, *n*=17) and *C587*^{ts}>*Madm*^{RNAi} (**c,d**, 2 days at 29°C, *n*=25) were immunostained with Vasa (red), βPS-integrin (red) and DAPI (blue). (**e**) The testes containing *C587*^{ts}>Control (**e,** *n*=12) and *C587*^{ts}>*Madm*^{RNAi} (**f**, n=14) 2 days at 29°C, were immunostained with DE-Cadherin (green), βPS-integrin (red) and DAPI (blue). Integrin is dramatically increased in the *C587*^{ts}>*Madm*^{RNAi} testis at the CySCs–hub interface. White arrows indicate GSC. CySCs are highlighted by yellow arrowheads (**a,e**). Pink arrows indicate integrin localization (**a-f**). Asterisks indicate hub cells. (**g**) A bar graph showing the quantitation of fluorescence intensity of βPS-integrin in *C587*^{ts}>Control and *C587*^{ts}>*Madm*^{RNAi} testes, 2 days at 29°C. All values are mean ± s.e.m. Statistical significance determined by Student's *t*-test, ****P*<0.0001. Scale bars (**a–f**)**:** 10 μm.

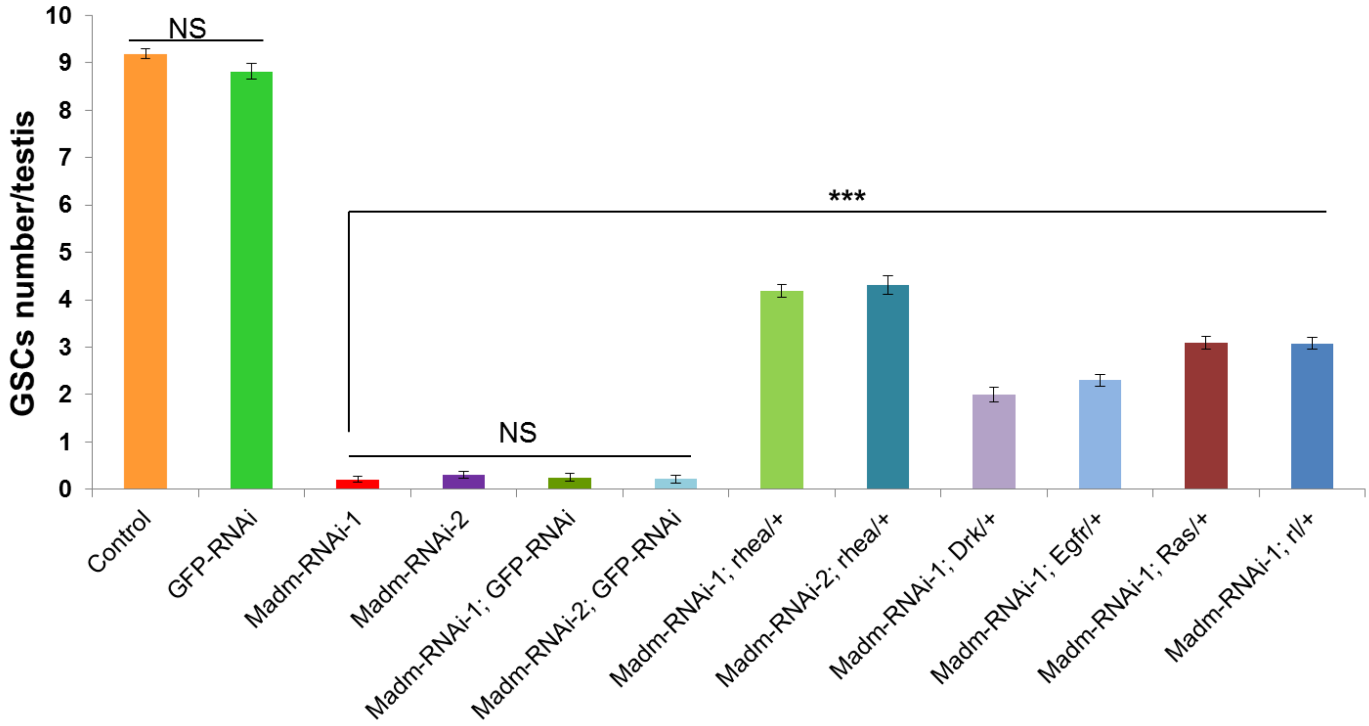

Supplementary Figure 9 | Madm prevents CySCs from outcompeting GSCs via integrin and the EGFR pathway. The Quantification of the number of GSCs attached to hub cells. At 7 days at 29°C, GSCs in the testes of *C587*^{ts}>*Madm*$^{RNAi-1}$ and *C587*^{ts}>*Madm*$^{RNAi-2}$ dramatically decrease compared to the *C587*^{ts}>Control testes (*P*<0.0001). Removing one copy of *rhea*$^{6-66}$, *rhea*$^{13-8}$, or reducing the dosage of the EGFR pathway components, *Drk*^{TZ160}, *Egfr*^{f24}, *Ras*^{C40b} and *rl*^{698}, significantly rescued the phenotypes associated with the *Madm*^{RNAi} phenotype. All values are mean ± s.e.m. Statistical significance determined by Student's *t*-test (between control and *GFP*^{RNAi}), NS, not significant; Statistical significance determined by one-way analysis of variance, ****P*<0.0001.

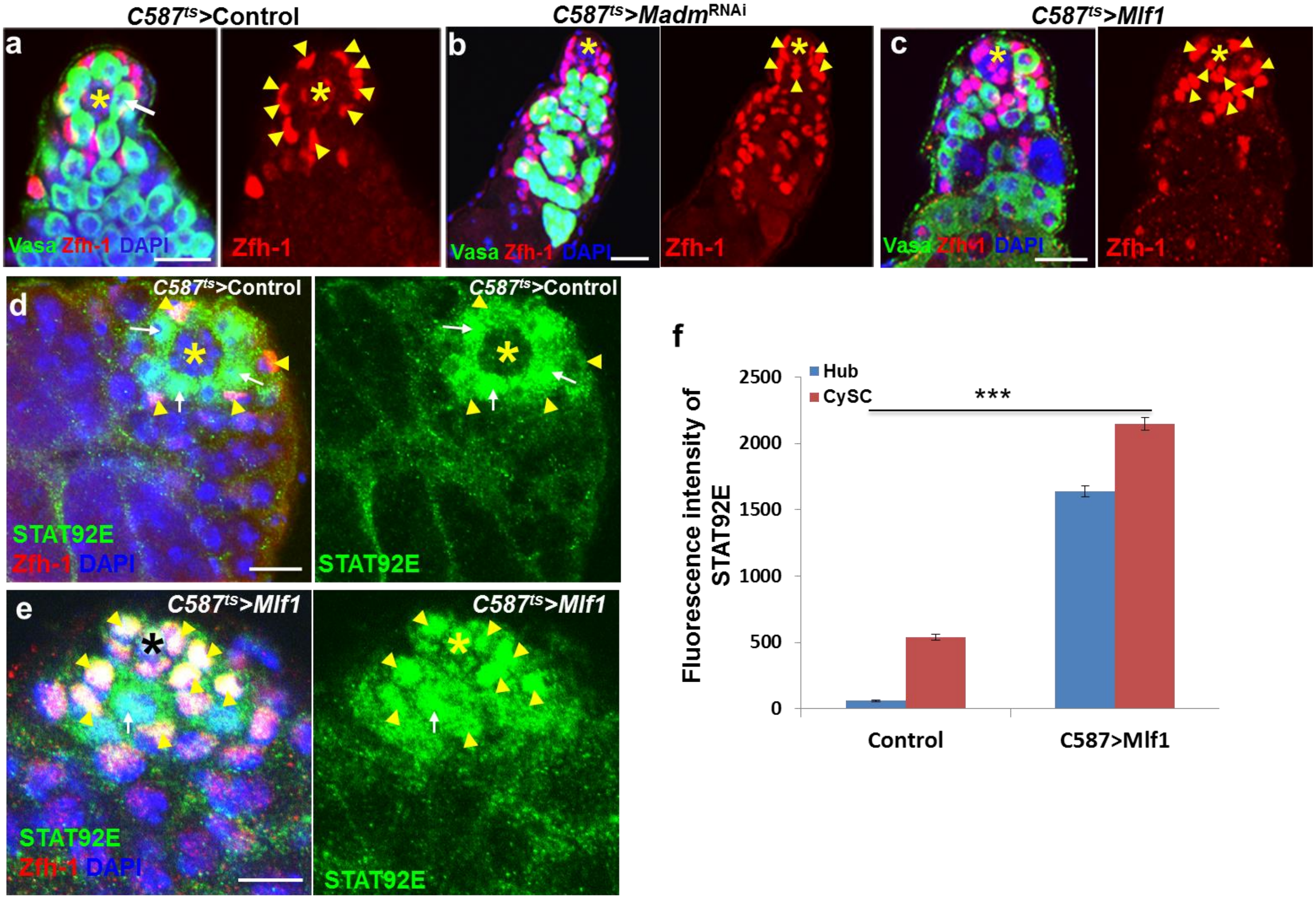

Supplementary Figure 10 | Mlf1 and Madm play opposite roles in regulating stem cell competition. (a–c) The testes containing *C587*^{ts}>Control (**a**, 5 days at 29°C, *n*=20), *C587*^{ts}>*Madm*^{RNAi} (**b**, 2 days at 29°C, *n*=17) and *C587*^{ts}>*Mlf1* (**c**, 5 days at 29°C, *n*=25). The testes were immunostained with antibodies against Vasa (green) and Zfh-1 (red). (**d,e**) Testes of *C587*^{ts}>Control (**d**, 5 days at 29°C, *n*=24) and *C587*^{ts}>*Mlf1* (**e**, 5 days at 29°C, *n*=20). The testes were immunostained with Stat92E (green) and Zfh-1 (red) and DAPI (blue). White arrows indicate GSC. CySCs are highlighted by yellow arrowheads. (**f**) A bar graph showing the quantitation of fluorescence intensity of Stat92E in hub and CySCs in *C587*^{ts}>Control and *C587*^{ts}>*Madm*^{RNAi} testes, 5 days at 29°C. All values are mean ± s.e.m. Statistical significance determined by Student's *t*-test, ***P<0.0001. Asterisks indicate hub cells. Scale bars (**a–e**)**:** 10 μm.

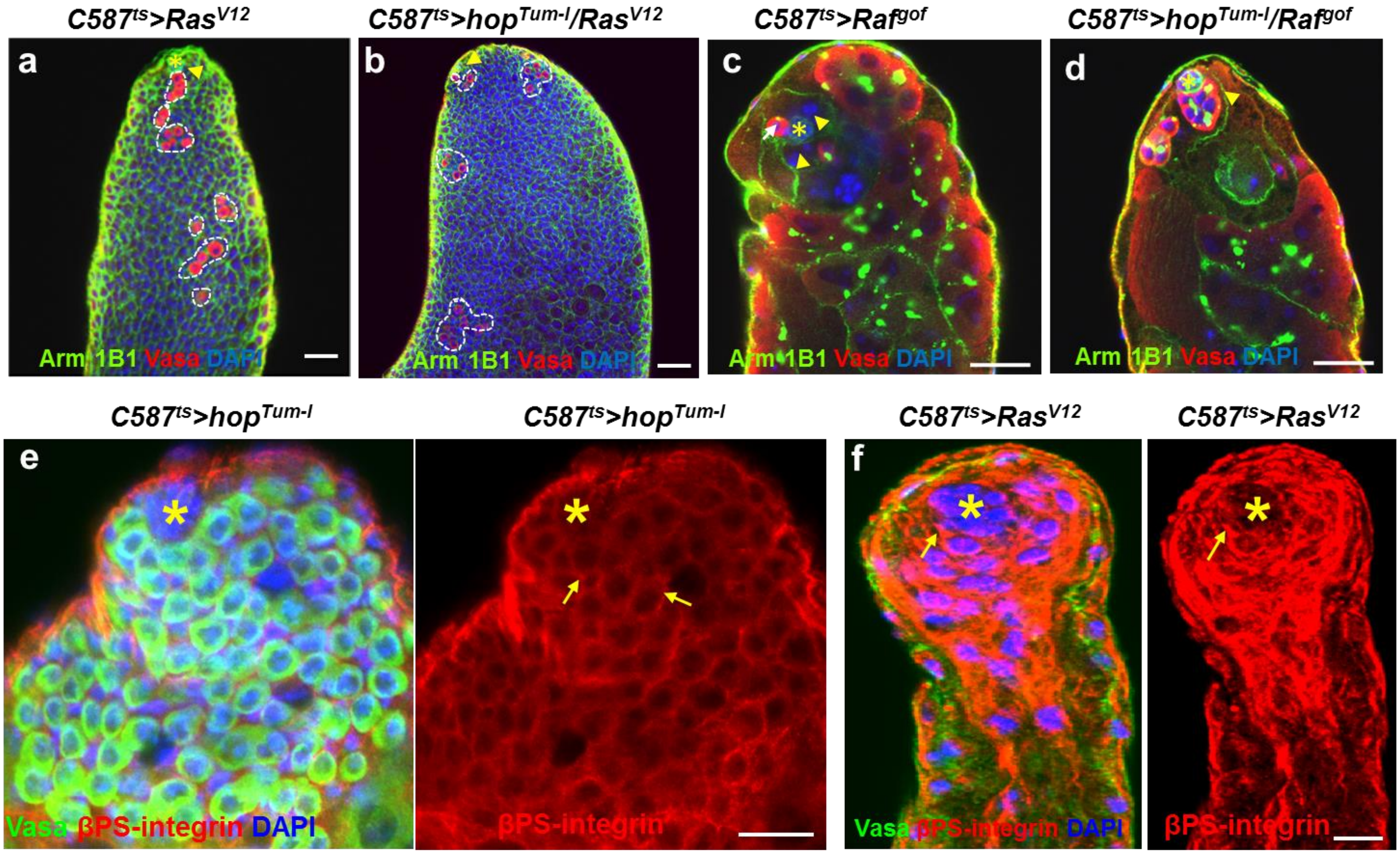

Supplementary Figure 11 | The EGFR/Ras/Raf/ERK pathway functions downstream of the JAK pathway in regulating CySC and GSC competition at the testis niche. (**a–d**) GSCs in the testes of *C587*^{ts}>*Ras*^{V12} (**a**, *n*=17), *C587*^{ts}>*hop*$^{Tum-l}$/*Ras*^{V12} (**b**, *n*=22), *C587*^{ts}>*Raf*^{gof} (**c**, *n*= 20), and *C587*^{ts}>*hop*$^{Tum-l}$/*Raf*^{gof} (**d**, *n*= 25). Testes were immunostained after 7 days culturing at 29°C with Vasa (red), 1B1 and Arm (green) and DAPI (blue). White arrows indicate GSC. CySCs are highlighted by yellow arrowheads. **(e,f)** The testes of *C587*^{ts}>*hop*$^{Tum-l}$ (**e**, *n*=17), and *C587*^{ts}>*Ras*^{V12} (**f**, *n*=25). The testes were cultured for 6 days at 29°C before they were immunostained with Vasa (green), βPS-integrin (red), and DAPI (blue). Yellow arrows indicate integrin localization. (**e,f**) Asterisks indicate hub cells. Scale bars (**a–f**)**:** 10 μm.

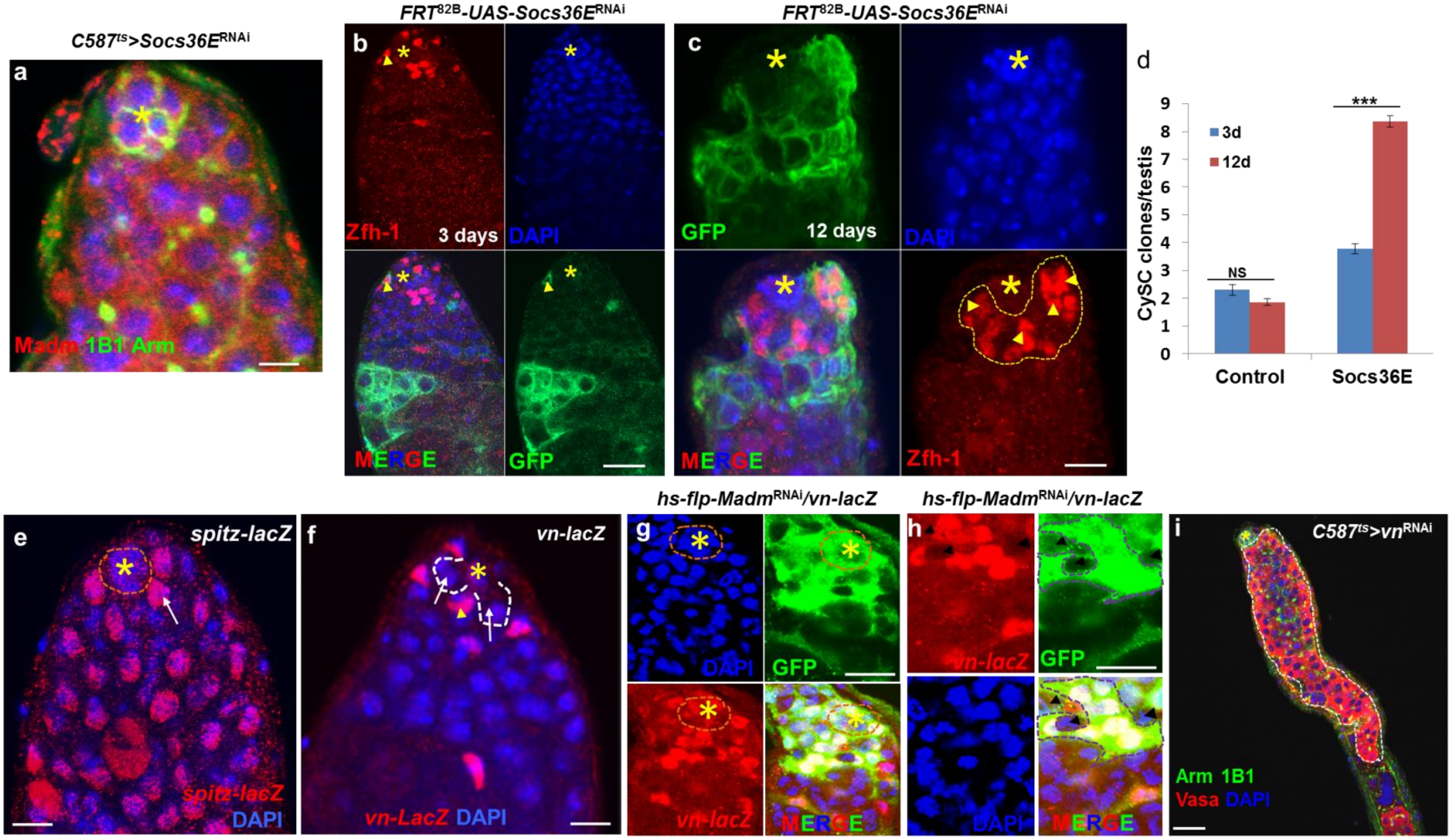

Supplementary Figure 12 | Socs36E cell–autonomously prevents CySCs from outcompeting GSCs and Madm negatively regulates the EGFR signaling pathway by repressing *vn* expression. (a) The testes of *C587*^{ts}*Socs36E*^{RNAi} were stained with Madm (red), 1B1 and Arm (green), DAPI (blue). **(b and c)** GFP positive clones were generated in the testes of *FRT*^{82B}*-UAS-Socs36E*^{RNAi} flies using the MARCM technique and were stained at 3 days (**b**, *n*=27) and 12 days (**c**, *n*=30) ACI with Zfh-1 (red), GFP (green), and DAPI (blue). The number of GFP-positive CySCs (Zfh-1+ cells, **c**) increased at 12 days ACI and moved to the tip of the testes, compared to 2 days ACI (**b**). CySCs are highlighted by yellow arrowheads. (**d**) A bar graph showing the quantitation of CySC clones per testis in *FRT*^{82B}-Control and *FRT*^{82B}-*UAS-Socs36E*^{RNAi} flies at 3 days and 12 days ACI. (**e**) The testes of *spitz-LacZ*-Control were immunostained with β-galactosidase (red) and DAPI (blue). **(f)** The testes of *vn-LacZ*-Control (*n*=12) were immunostained with β-galactosidase (red) and DAPI (blue). White arrows indicate GSC. CySCs are highlighted by yellow arrowheads. The testes of *hs-flp-Madm*^{RNAi}*/vn-lacZ* (**g,h**; *n*=35) were immunostained with β-galactosidase (red), GFP green, and DAPI (blue). *vn-LacZ* expression is significantly increased in GFP+ flip-out CySC clones (**h**, purple dotted lines), compared to wild type GFP-negative cells (**h,** black arrowhead). (**i**) GSCs in the testes containing *C587*^{ts}*>vn*^{RNAi} (*n*=32), were stained after culturing for 7 days at 29°C with Vasa (red), 1B1 and Arm (green) and DAPI (blue). White dotted lines representing GSC tumor phenotype (**i**). Asterisks indicate hub cells. All values are mean ± s.e.m. Statistical significance determined by Student's *t*-test ****P*<0.0001; NS, not significant. Scale bars (**a-c, e-i**): 10 μm.

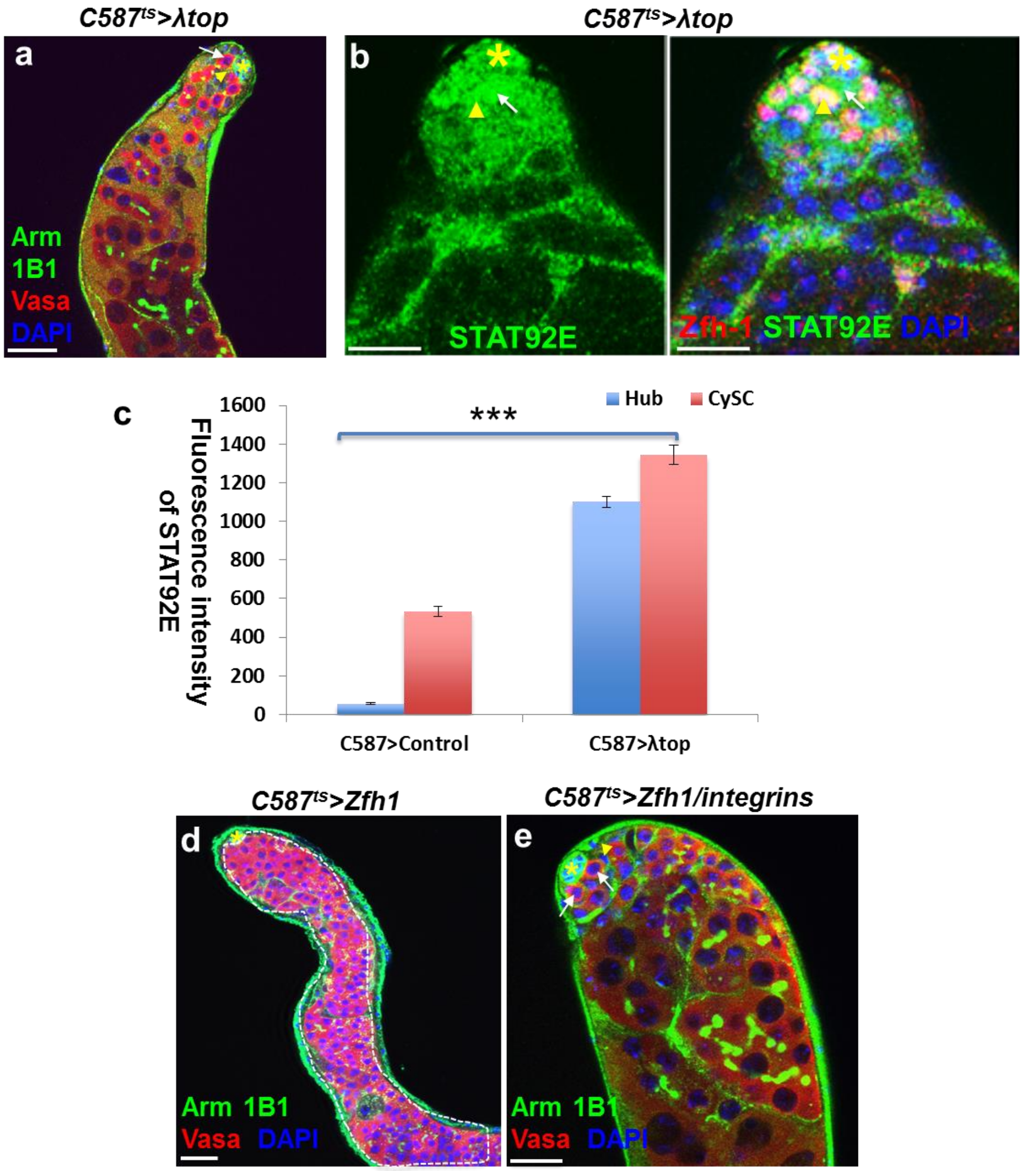

Supplementary Figure 13 | Expressing constitutive active form of EGFR enhances Stat92E expression in CySCs and overexpression of integrin rescued GSC tumor phenotypes associated with overexpression of *Zfh1*. **(a)** GSCs in the testes of *C587*^{ts}>*λtop* (*n*=24) were stained after 7 days culturing at 29°C with Vasa (red), 1B1, Arm (green) and DAPI (blue). (**b**) Confocal sections through the apex of the testes containing *C587*^{ts}>*λtop* (*n*=20) were stained with Stat92E (green) and Zfh-1 (red) and DAPI (blue). Stat92E expression is increased in CySCs (Zfh-1+ cells) and hub cells. (**c**) A bar graph showing the quantitation of fluorescence intensity of Stat92E in hub and CySCs in *C587*^{ts}>Control and *C587*^{ts}>*λtop* testes. All values are mean ± s.e.m. Statistical significance determined by Student's *t*-test, ****P*<0.0001. **(d,e)** GSCs in the testes containing *C587*^{ts}>*UAS-Zfh1* (D, *n*=17) and *C587*^{ts}>*Zfh1/PS1 ßPS(integrins)* (*n*=25) were stained after culturing for 7 days at 29°C with Vasa (red), 1B1, Arm (green) and DAPI (blue). White dotted lines representing GSC tumor phenotype. (**d**). White arrows indicate GSC. CySCs are highlighted by yellow arrowheads (**a,b,e**). Asterisks indicate hub cells. Scale bars (**a,b,d,e**)**:** 10 µm.

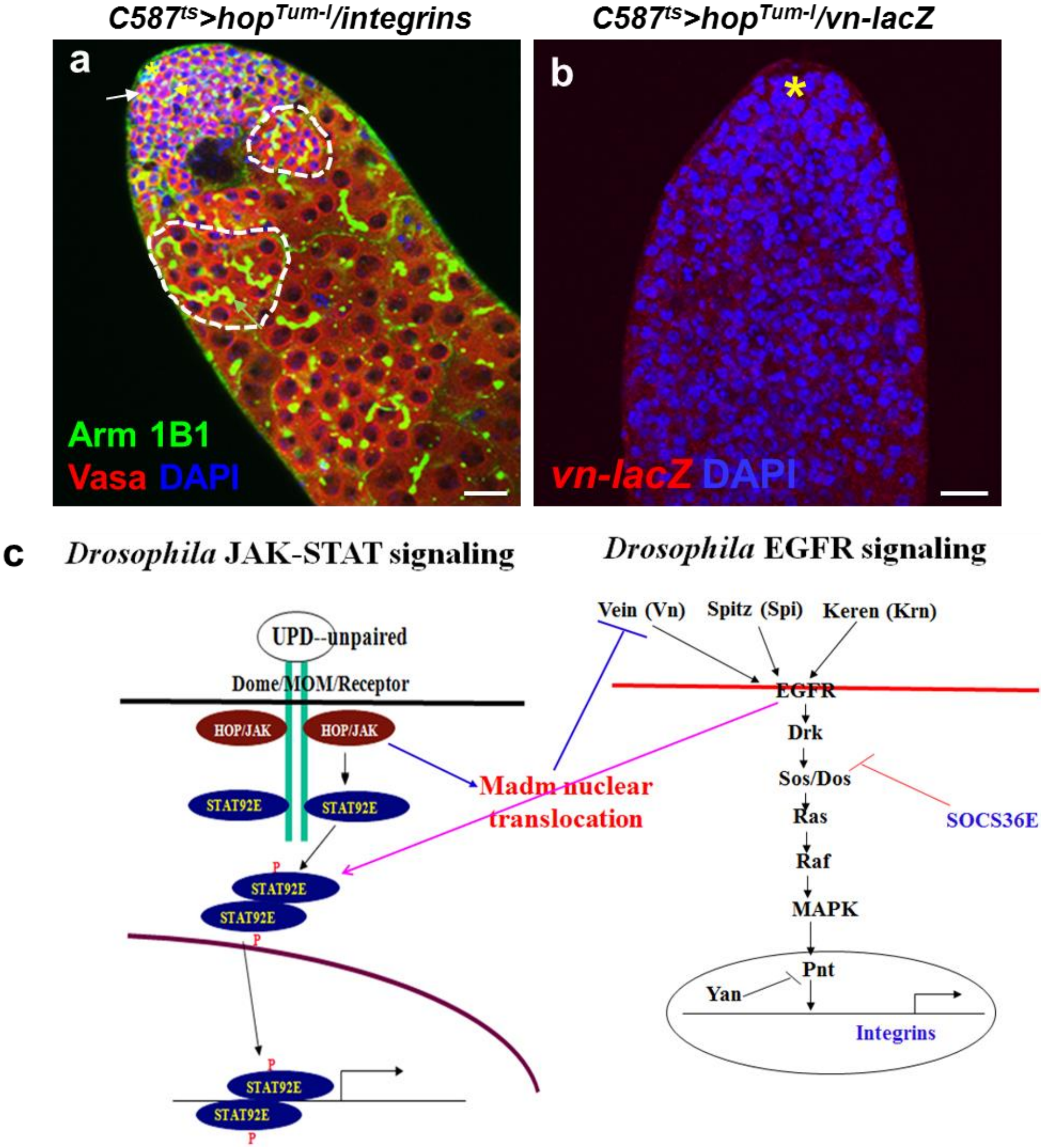

Supplementary Figure 14 | A model of how Madm regulates GSC and CySC competition in *Drosophila* testis. (**a**) GSCs in the testes containing *C587*^{ts}*>hop*$^{Tum\text{-}l}$*/PS1 ßPS(integrins)* (*n*=27) were stained after cultured 7 days of culturing at 29°C with Vasa (red), 1B1, Arm (green), and DAPI (blue). White arrows indicate GSC. CySCs are highlighted by yellow arrowheads. White dotted lines representing differentiated cells. (**b**) The testes of *C587*^{ts}*>hop*$^{Tum\text{-}l}$*/vn-LacZ* (*n*=31, 7 days at 29°C) were immunostained with β-galactosidase (red) and DAPI (blue). Scale bars (**a,b**): 10 μm. (**c**) A model of how Madm regulates GSC and CySC competition in *Drosophila* testis. Details are described in the text.

Supplementary Note 1

Generating *Madm* mutant GSCs clones. Following genotypes were used to generate *Madm* mutant GSCs clones:

+/SM6, hs-Flp; FRT^{82B}*- Madm*^{3G5}*/ FRT*^{82B}*-Arm-lacZ*

+/SM6, hs-Flp/+; FRT^{82B}*- Madm*^{7L2}*/ FRT*^{82B}*-Arm-lacZ*

MARCM clonal analysis. To generate GFP-marked mutant CySC clones using the MARCM system, flies with the following genotypes were used:

UAS-mcD8-GFP hs-Flp /y; UAS-hop^{Tum-l}*/+; tub-Gal4-FRT*^{82B}*-tub-Gal80/FRT*^{82B}*-PiM*

UAS-mcD8-GFP hs-Flp /y; UAS-Ras^{V12}*/+; tub-Gal4-FRT*^{82B}*-tub-Gal80/FRT*^{82B}*-PiM*

UAS-mcD8-GFP hs-Flp /y;UAS-Socs36E^{RNAi}*/+;tub-Gal4-FRT*^{82B}*-tub-Gal80/FRT*^{82B}*-PiM*

UAS-mcD8-GFP hs-Flp /y;SM6,hs-Flp/+/+;tub-Gal4-FRT^{82B}*-tub-Gal80/FRT*^{82B}*- PiM*

UAS-mcD8-GFPhs-Flp/y;SM6,hs-Flp/+/+;tub-Gal4-FRT^{82B}*-tub-Gal80/FRT*^{82B}*-Madm*^{3G5}

UAS-mcD8-GFPhs-Flp/y;SM6,hs-Flp/+/+;tub-Gal4-FRT^{82B}*-tub-Gal80/FRT*^{82B}*-Madm*^{7L2}

Sense oligo for the transgenic RNAi lines:

<u>VDRC lines</u>

V35641 (*rl*): 5’-CGCGAATTCTTGCGACTTTGGATTGGCTCGT-3’
V51821 (*Socs36E*): 5’-CGCGAATTCACGCCAGCCATCACCATC-3’
V27346 (*Madm*): 5’CGCGAATTCCCATCCAGCACCACCCTTCC-3’

<u>Bloomington lines</u>

BL31644 (*Madm*): 5’-AGAATTCAATAAAAACAATACGCGCCG-3’
BL42529 (*Madm*): 5’-CCCGTAGTGGATACCACGAAA-3’
BL41599 (*Madm*): 5’-CAGCGAGTCAACTGCCATCAA-3’

<u>NIG line</u>

10491R-2(*vn*):5’-AAGGCCTACATGGCCGGACCGTGGGAAGGCAGTGCCGAAGAA-3’
